# Evaluation of measures of sustainability and sustainability determinants for use in community, public health, and clinical settings: a systematic review

**DOI:** 10.1186/s13012-022-01252-1

**Published:** 2022-12-13

**Authors:** Alix Hall, Adam Shoesmith, Emma Doherty, Brydie McEvoy, Kayne Mettert, Cara C. Lewis, Luke Wolfenden, Serene Yoong, Melanie Kingsland, Rachel C. Shelton, Shannon Wiltsey Stirman, Noor Imad, Rachel Sutherland, Nicole Nathan

**Affiliations:** 1 School of Medicine and Public Health, The University of Newcastle, Locked Bag 10 Wallsend, Callaghan, NSW Australia; 2 Priority Research Centre for Health Behaviour, The University of Newcastle, Callaghan, NSW Australia; 3 Hunter Medical Research Institute, New Lambton Heights, New South Wales Australia; 4 Hunter New England Population Health, Hunter New England Local Health District, Wallsend, NSW Australia; 5 Kaiser Permanente Washington Health Research Institute, Seattle, USA; 6 Department of Psychology, University of Washington, Seattle, USA; 7 School of Health Sciences and Social Development, Deakin University, Melbourne, Victoria Australia; 8 Mailman School of Public Health, Department of Sociomedical Sciences, Columbia University, New York, New York USA; 9 Dissemination and Training Division, National Center for PTSD and Department of Psychiatry and Behavioural Sciences, Stanford Medicine, Stanford University, Palo Alto, California USA; 10 School of Health Sciences, Department of Nursing and Allied Health, Swinburne University of Technology, Hawthorn, Victoria Australia

**Keywords:** Sustainability, Determinants, Psychometrics, Measurement

## Abstract

**Background:**

Sustainability is concerned with the long-term delivery and subsequent benefits of evidence-based interventions. To further this field, we require a strong understanding and thus measurement of sustainability and what impacts sustainability (i.e., sustainability determinants). This systematic review aimed to evaluate the quality and empirical application of measures of sustainability and sustainability determinants for use in clinical, public health, and community settings.

**Methods:**

Seven electronic databases, reference lists of relevant reviews, online repositories of implementation measures, and the grey literature were searched. Publications were included if they reported on the development, psychometric evaluation, or empirical use of a multi-item, quantitative measure of sustainability, or sustainability determinants. Eligibility was not restricted by language or date. Eligibility screening and data extraction were conducted independently by two members of the research team. Content coverage of each measure was assessed by mapping measure items to relevant constructs of sustainability and sustainability determinants. The pragmatic and psychometric properties of included measures was assessed using the Psychometric and Pragmatic Evidence Rating Scale (PAPERS). The empirical use of each measure was descriptively analyzed.

**Results:**

A total of 32,782 articles were screened from the database search, of which 37 were eligible. An additional 186 publications were identified from the grey literature search. The 223 included articles represented 28 individual measures, of which two assessed sustainability as an outcome, 25 covered sustainability determinants and one explicitly assessed both. The psychometric and pragmatic quality was variable, with PAPERS scores ranging from 14 to 35, out of a possible 56 points. The Provider Report of Sustainment Scale had the highest PAPERS score and measured sustainability as an outcome. The School-wide Universal Behaviour Sustainability Index-School Teams had the highest PAPERS score (score=29) of the measure of sustainability determinants.

**Conclusions:**

This review can be used to guide selection of the most psychometrically robust, pragmatic, and relevant measure of sustainability and sustainability determinants. It also highlights that future research is needed to improve the psychometric and pragmatic quality of current measures in this field.

**Trial registration:**

This review was prospectively registered with Research Registry (reviewregistry1097), March 2021.

**Supplementary Information:**

The online version contains supplementary material available at 10.1186/s13012-022-01252-1.

Contributions to the literature

- Sustainability is concerned with the continued use and benefit of effective interventions.
- Measuring sustainability is a complex issue, with the number of measures increasing and measurement quality variable.
- Currently, there is no comprehensive evaluation of sustainability measures and their quality across a wide range of settings.
- This review provides an extensive review and evaluation of the quality of available measures of sustainability and sustainability determinants across a broad range of contexts.
- The results can be used to guide the selection of the most robust and relevant measure of sustainability and sustainability determinants. It also highlights where additional research is warranted.

## Introduction

Maintaining the delivery and health impact of evidence-based interventions (EBIs) over time is a challenge across a range of community, public health, and clinical settings [1–3]. A 2020 systematic review of 18 multi-component school-based public health interventions found that none of the interventions continued to be delivered in their entirety (i.e., all components) once active implementation support (i.e., provision of start-up funding and/or other resources) ceased [4]. Similarly, only seven of 18 evaluations sustained clinical practice guidelines in a variety of healthcare settings following active implementation in a recent systematic review [5]. Understanding why EBI implementation attenuates over time, and how best to support their long-term delivery is necessary to ensure that implementation investments are worthwhile. This concept, referred to as “sustainability,” is an important outcome in implementation science [6].

Similar to other emerging fields, the definitions relating to concepts of sustainability have been varied and at times conflicting [7], emphasising the call for a nomenclature in this field. However, more recently a recommended definition of sustainability has been recognised as “the continued delivery of an innovation or intervention, potentially after adaptation, at a sufficient level to ensure the continued health impact and benefits of the intervention” [7]. While sustainability determinants are defined as “the characteristics or factors associated with the continued use and impact of an EBI” [8–10]. Several frameworks recognise and conceptualise the complex and dynamic nature of sustainability [2, 11–13]. The Integrated Sustainability Framework developed by Shelton and colleagues (2018) [2] outlines recommendations on how sustainability should be conceptualised and measured. It also organises influential multi-level factors (i.e., determinants) into five domains (i.e., outer context, inner context, intervention characteristics, processes, and implementer and population characteristics) [2, 14].

Central to any field is measurement validity, or the ability to accurately measure relevant concepts, outcomes, and constructs. To do this, a measure should comprehensively and adequately cover the intended construct. This is known as content validity [15] and is recognised as one of the most important measurement properties [16]. For measures of sustainability as an outcome to have adequate content validity, they should encompass the features of a multi-component definition, such as that proposed by Moore and reflect concepts of time, continued delivery of the EBI, maintained behaviour change, evolution and/or adaptation of the program, and continued health and other benefits [7]. Measures should also illustrate reliability and evidence of other domains of validity (e.g., concurrent validity), to ensure accuracy and reduce error. Finally, measures should exhibit important pragmatic qualities, including easy access, use, scoring, and interpretation [17]. Pragmatic qualities are less frequently evaluated but are essential in ensuring the uptake of reliable and valid measures.

Identifying and measuring sustainability, as well as factors related to sustainability (i.e., determinants), is complex given the diverse and dynamic settings being studied. Consequently, many existing measures have only been used once [18], illustrating limited standardisation in measurement. This makes it difficult to compare and synthesise findings across studies. Furthermore, there has been a lack of distinction between measures of sustainability determinants and sustainability as an outcome [2, 9].

High-quality systematic reviews on available measures, their psychometric and pragmatic properties, and how they have been empirically used are essential for providing evidence-based recommendations on which measures to use, identifying gaps in measurement and highlighting areas for future research [19]. There are two systematic reviews exploring measures of sustainability as an implementation outcome in health care settings focused on mental health and substance use [18, 20]. Overall, psychometric assessment reporting was poor, with only one psychometric indicator; norms, reported in more than half of the identified sustainability measures. They also found that most (54%) measures were used only a single time. While these two reviews provide a thorough evaluation of sustainability measures, they were limited by a narrow focus on behavioral health settings and a subset of psychometric and pragmatic properties. A third review, by Moullin et al. [8], used snowball sampling to identify sustainment and sustainability measures across a broader range of community, public health, and clinical settings, offering general guidance about how and in what circumstances each measure could be used, but no formal assessment of their quality was undertaken.

Collectively, these three reviews offer an excellent foundation for informing a comprehensive systematic review and critical assessment of both the psychometric and pragmatic qualities of measures of sustainability (as an outcome) and sustainability determinants, across a range of settings. This review addresses important gaps by allowing researchers to identify where robust and suitable measures exist, to reduce unnecessary duplication, and provide practical guidance to end-users in selecting the most relevant measure for their setting.

Specifically, we aimed to:

1. Assess content validity by mapping the constructs covered by identified measures of: (a) sustainability as an outcome to the multidimensional definition of sustainability proposed by Moore et al. [7]; and (b) sustainability determinants to the domains and constructs outlined by the Integrated Sustainability Framework [2]
2. Assess the psychometric and pragmatic qualities of identified measures using a standardised assessment tool
3. Describe how each of the identified measures have been applied in empirical research.

## Methods

This systematic review is reported according to the Preferred Reporting Items for Systematic review and Meta-Analysis Protocols checklist (PRISMA) [21] (see Additional file 1) and followed established procedures used by other systematic reviews of measures of implementation outcomes [18, 20, 22, 23]. It was registered prospectively with Research Registry (reviewregistry1097) prior to the final database search being conducted.

### Search strategy

An extensive search strategy, informed by previous reviews of implementation measures [18, 24–27] and reviews on sustainability determinants [14], was employed to identify eligible measures of sustainability and sustainability determinants. We searched the following electronic databases on 6 of June 2021: the Cochrane Central Register of Controlled trials (CENTRAL), MEDLINE, EMBASE, PsycINFO, ERIC, CINHAL, and SCOPUS. The search included keywords relevant to the three levels of search terms: (i) terms relevant or synonymous with the constructs of interest, sustainability, and sustainability determinants (e.g., sustain*, implement*); (ii) psychometric properties (e.g., psychometric*, reliab*); and (iii) setting (e.g., public health, evidence-based medicine). Please see Additional file 2: Table S1a to S1G for an example of the search strategy. Similar to previous reviews, we defined a measure as a multi-item survey, questionnaire, instrument, tool, or scale [24] that is quantitatively scored. Reference lists of previous relevant reviews were also searched. New measures published outside our search date and identified through journal alerts and snowball searching were also included. For aims 1 and 2, only full-text articles were eligible for inclusion. The authors of conference abstracts were contacted to obtain full-text articles.

Online repositories of implementation measures, including the “Society for Implementation Research Collaboration Instrument Repository” [28] and the “Dissemination & Implementation Models in Health Research and Practice” [29] web tool, were also searched. Finally, a forward literature search was undertaken for each relevant measure, whereby two researchers independently searched the name of identified measures within Google Scholar. The first 100 hits were checked for relevance or until relevant articles were no longer being identified. A citation search of the original development paper for each measure was conducted to identify empirical studies that used each measure. For measures that did not have a specified name, only the citation search was conducted. These searches were conducted independently by pairs of researchers (either BM, AH, CG, SH, or KA) between April 2021 and May 2022. For the third aim, published scientific manuscripts, reports, abstracts, trial registrations, and protocol papers describing the empirical use of eligible measures were included.

### Inclusion/exclusion criteria

Publications were included if they reported on the development, psychometric evaluation, or empirical use of a multi-item, quantitative, self-report measure that is scored, of sustainability as an outcome or sustainability determinants, designed to be used in a community, public health, or clinical setting. Individual measures were the unit of interest as the development and psychometric evaluation of measures are usually reported across multiple publications. Empirical studies that applied the identified measures were included, to allow for an evaluation of how identified measures have been used in the field. Only measures that assumed a reflective measurement model of sustainability or sustainability determinants were included (i.e., consist of items that sought to reflect the underlying construct of sustainability or sustainability determinants and did not alter or define the construct such as an index) [26]. Publications of any language were included, and wherever possible, non-English publications were translated via colleagues or contacts proficient in the language of interest or Google translate. No restrictions were made on health condition or the target population. Published or unpublished full-text articles or papers were eligible. We excluded measures that were based on a formative measurement model (i.e., items define the underlying construct such as an index), as such measures were not relevant to the constructs we were assessing, and different properties are used to assess their rigor. Unscored checklists and single item tools were excluded, as these serve a different purpose than measures designed to quantify an underlying construct. Measures designed explicitly for a specific study and not for wider use in the field (i.e., one-time use measures) were excluded, as were qualitative measures.

### Study selection

The search results from the electronic databases were managed and duplicates identified using EndNote version X9.2 software (Thomson Reuters, PA, and U.S.) The de-duplicated library was imported into Covidence [30], where article screening occurred. Both title and abstract and full-text screening were conducted independently by two members of the research team (either AS, BM, AH, NN, NI, NM, or KA). Conflicts were resolved by a third member of the research team (AH or AS).

### Critical assessment

The pragmatic and psychometric evidence of each eligible measure was assessed and scored using the Psychometric and Pragmatic Evidence Rating Scale (PAPERS) [17, 31]. PAPERS includes 14 items that assesses nine psychometric properties and five pragmatic features (see Table 1). Each item is scored using a six-point Likert scale ranging from −1 (poor) to 4 (excellent) [17, 31]. The PAPERS criteria were applied to each individual measure, rather than an individual study or publication, as multiple publications often report on different aspects of a measure’s pragmatic and psychometric properties. For measures that had multiple reports of the same pragmatic or psychometric property, for instance in the case of multiple studies assessing the responsiveness, the median score was used. If the median value resulted in a non-integer, the score was rounded down [18, 23, 27]. Data were only assessed against the PAPERS psychometric criteria if it was being explicitly used to evaluate the psychometric properties of that measure. Due to the typically poor reporting of pragmatic indicators of a measure, grey literature, such as scoring manuals, were reviewed to assess such qualities. The quality of empirical studies was not assessed, as we were only interested in describing the application and use of eligible measures, aspects which are not influenced by the rigour of the research design or potential bias.

**Table 1:** Characteristics of measures of sustainability and sustainability determinants and overall psychometric and pragmatic quality scores

Measure name	Alternate name	Designed explicitly for Sustainability	Other constructs covered	Theory/framework	Target population involved in development	Setting/s developed and validated in	Country validated in and languages available	EBI and setting designed to assess^d^	User perspective	Total PAPERS’ scores^**a**^
**Measures of sustainment**										
**Provider Report of Sustainment Scale (PRESS)** [32]	None	Yes	None	No	Yes	Substance use disorder treatment, child welfare, education, and specialty mental health	**Country:** USA **Language:** English	General EBI and setting	Executives and frontline staff	**35**
**Stages of implementation completion (SIC) – Original** [33–35]	None	No	Implementation process/stages	Unclear	Yes	Multiple	**Country:** USA **Language:** English	General EBI and setting	Researcher/purveyor	**14**
**Measures of sustainability determinants**										
**Assessment of Barriers to Implementation and Sustainability in Schools (ABISS)** [36]	None	Yes	None	Framework proposed by the authors.	No	School	**Country:** USA **Language** English	Specific EBI: School-wide Positive Behavior Support (SWPBS) Specific setting: school	Executive staff, frontline staff, and external personnel who have knowledge of a school's SWPBS systems.	**26**
**Advanced Level Tier Interventions Treatment Utilization and Durability (ALTITUDE)** [37]	None	Yes	None	No	Yes	School	**Country:** USA **Language:** English	Specific EBI: School-wide Positive Behavior Support (SWPBS) implementation at Tiers 1, 2, and 3 Specific setting: schools	Executive staff, frontline staff, and external personnel who have knowledge of a school's SWPBS systems.	**21**
**A measurement instrument for sustainability of work practices in in long-term care - long version** [38]	None	Yes	None	Framework proposed by the authors.	Yes	Nursing homes, elderly homes, home care and care for disabled	**Country:** Netherlands **Language:** Dutch English	General EBI and setting	Executive and frontline staff	**26**
**A measurement instrument for sustainability of work practices in in long-term care - short version** [38]	None	Yes	None	Framework proposed by the authors.	Yes	Nursing homes, elderly homes, home care and care for disabled	**Country:** Netherlands **Language:** Dutch English	General EBI and setting	Executive and frontline staff	**22**
**Change Process Capability Questionnaire (CPCQ)** [39]	None	No	Change management	Framework proposed by the authors on organisational change	Yes	Medical groups treating patients with depression	**Country:** USA **Language:** English	Specific EBI: Depression care Specific: Clinical/healthcare setting	Executive staff	**19**
**Clinical Sustainability Assessment Tool (CSAT)** [40]	None	Yes	None	Unclear	Yes	Multiple clinical settings	**Country:** USA **Language:** English	General EBI Specific setting: Clinical/healthcare setting	Executive and frontline staff	**28**
**Faith-Based Organization Health Integration Inventory (FBO-HII)** [41]	None	Yes	None	No	Yes	African American Churches	**Country:** USA **Language:** English	Specific EBI: Health promotion activities Specific setting: Faith based organisations	Executive staff	**17**
**General Organizational Index (GOI)** [42, 43]	None	No	Quality of implementation.	No	Yes	Community mental health centers	**Country:** Norway USA **Language:** English Norwegian	General EBI and setting	Researcher/purveyor	**24**
**Levels of Institutionalization (Loln)** [44, 45]	None	Yes	None	Level of Institutionalization Matrix framework [46]	Yes	Organisations that operate health promotion programs - public schools, county health departments, and non-profit health agencies Hospitals and home health agencies	**Country:** USA **Language:** English	Specific EBI: Health promotion programs General setting	Executive staff	**25**
**National Health Service (NHS) Sustainability Model and Guide** [47]	The National Health Service (NHS) Institute for Innovation and Improvement Sustainability Model	Yes	None	A broader sustainability model proposed by the authors	Yes	Health care settings	**Country:** UK **Language:** English	General EBI Specific setting: clinical settings	Executive and frontline staff	**17**
** The Normalisation Measure Development questionnaire (NoMAD)** [48–53]	None	Yes	None	Normalization Process Theory [54]	Yes	Multiple clinical and community settings	**Country:** UK USA Netherlands Canada Sweden Brazil **Language:** Albanian Danish Dutch English French German Italian Norwegian Portuguese Spanish Swedish	General EBI and setting	Executive and frontline staff	**22**^**b**^
**New South Wales Sustainability Checklist** [55]	None	Yes	None	None	Unclear	Health promotion programs	**Country:** Australia **Language:** English	Specific EBI: Health promotion workers General setting	Executive and frontline staff	**16**
**Office of Adolescent Health (OAH) Sustainability Assessment** [56]^**c**^	None	Yes	None	The OPA Framework for Program Sustainability	Unclear	Adolescent health promotion and disease prevention	**Country:** USA **Language:** English	General EBI and setting	Executive and frontline staff	**16**
**Office of Population Affairs (OPA) Sustainability Assessment Tool** [57]^**c**^	None	Yes	None	The OPA Framework for Program Sustainability	Unclear	Adolescent health promotion and disease prevention	**Country:** USA **Language:** English	General EBI and setting	Executive and frontline staff	**18**
**Prevention Program Assessment** [58]	None	No	Organizational readiness for practice or program	Multiple theories	Yes	Public health departments, community organizations	**Country:** USA **Language:** English	Specific EBI: Chronic disease prevention programs General setting	Executive and frontline staff	**27**
**Program Sustainability Assessment Tool (PSAT)** [10]	None	Yes	None	Public Health Program Capacity for Sustainability framework [13]	Yes	State and community settings	**Country:** USA **Language:** English	Specific EBI: public health practice and programs General setting	Executive and frontline staff	**26**
**Program Sustainability Assessment Tool (PSAT) – adapted for elementary setting** [59]	None	Yes	None	Public Health Program Capacity for Sustainability framework [13]	Yes	School	**Country:** Australia **Language:** English	Specific EBI: public health practice and programs Specific setting: school	Executive and frontline staff	**19**
**Program Sustainability Index** [60]	None	Yes	None	Framework of sustainability proposed by the original authors.	Yes	Community settings	**Country:** USA **Language:** English	Specific EBI: Community-based programs General setting	Frontline staff	**23**
**School-wide Universal Behavior Sustainability Index- School Teams (SUBSIST)** [61–65]	None	Yes	None	Model of sustainability of school-based practices proposed by the original authors.	No	School	**Country:** USA **Language:** English	Specific EBI: School-wide Positive Behavior Support (SWPBS) implementation at Tiers 1, 2, and 3 Specific setting: schools	Executive staff, frontline staff and external personnel who have knowledge of a school's SWPBS systems.	**29**
**Sustainability Formative Self-Assessment Tool** [66]	None	Yes	None	The Georgia Health Policy Center Sustainability Framework	Unclear	Community rural health settings	**Country:** USA **Language:** English	General EBI and setting	Executive and frontline staff	**15**
**Sustainable Implementation Scale (SIS)** [67]	None	Yes	None	A range of frameworks covered by current systematic reviews.	No	National sites	**Country:** Sweden **Language:** English Swedish	Specific EBI: Community mental health services General setting	Researcher/purveyor	**23**
**Sustained Implementation Support Scale** [68]	None	Yes	None	No	Yes	Mix	**Country:** Australia, Barbados, Belgium, Canada, Germany, Ireland, Netherlands, New Zealand, Romania, UK, United Arab Emirates, USA **Language:** English	General EBI and setting	Executive and frontline staff	**23**
**Sustaining Innovation Through Education (SITE): Extended Behavioral** [69]	None	Yes	None	Author-derived framework	Yes	Schools	**Country** Australia **Language** English	General EBI Specific setting: schools	Executive and frontline staff	**18**
**Sustaining Innovation Through Education (SITE): short behavioral** [69]	None	Yes	None	Author-derived framework	Yes	Schools	**Country** Australia **Language** English	General EBI Specific setting: schools	Executive and frontline staff	**19**
**Sustainment Leadership Scale** [70]	None	Yes	None	Exploration, Preparation, Implementation, Sustainment (EPIS) framework [71]	Unclear	Community-based organizations	**Country:** USA **Language:** English	General EBI and setting	Frontline staff	**24**
**Sustainment Measurement System Scale (SMSS)** [72]*	None	Yes	None	Consolidated Framework for Implementation Research [73]	Yes	Substance Abuse and Mental Health Services	**Country:** USA **Language:** English	Specific EBI: prevention programs General setting	Executive and frontline staff	**28**

*Includes a sub-scale that assesses sustainability as an outcome and seven sub-scales assessing determinants of sustainability

^a^ Higher total PAPERS score reflects a higher level of psychometric and pragmatic quality, with the highest possible score being 56

^b^Total psychometric PAPERS rating for the Portuguese version of the NoMAD is 2, for the Swedish version (S-NoMAD) is 7, and for the Dutch version is 6, out of a possible 36. As the psychometric properties of translated measures may differ, the PAPERS ratings for these three translated versions of the NoMAD were scored separately

^c^The Office of Adolescent Health (OAH) and Office of Population Affairs (OPA) amalgamated. However, both measures developed by these divisions are still publicly available and contain different content, thus were assessed separately

^d^This is based on the intended focus of the measure and may differ to how the measure was originally developed and evaluated, which may reflect a more narrow focus than intended

### Data extraction

Data were extracted independently by two trained members of the research team (either NN, ED, AH, or AS), using a pre-piloted data extraction tool developed specifically for this study (Additional file 3). The data extraction form was programmed using REDCap; an electronic data capture tool hosted on the Hunter New England Population Health server [74, 75]. An overview of the main fields programmed in the data extraction tool are shown in Additional file 3.

To assess content coverage of the included measures, the items from each measure were mapped to constructs important to sustainability and sustainability determinants. For measures of sustainability (as an outcome), items were mapped to the five constructs outlined by Moore et al. [7] comprehensive definition of sustainability (see the “Introduction” section). Items from measures of sustainability determinants were first mapped to lower-level constructs that define five higher-level domains proposed by the Integrated Sustainability Framework  (i.e., outer context, inner context, intervention characteristics, processes, and implementer and population characteristics) [2] (see [14] for a more detailed description of the Integrated Sustainability Framework domains and constructs). Item mapping followed similar procedures undertaken in previous reviews [23, 76], whereby two research team members proficient in the content area of sustainability (AH & AS), independently extracted and mapped the items from each measure to the domains of the relevant frameworks outlined above. We classified a measure as incorporating components of a specific construct if at least one item was mapped to that construct. Discrepancies were resolved through discussion and input by two review members. We classified each measure as assessing either sustainability (as an outcome) or sustainability determinants based on the content of their items and which definition (see above) the items predominantly aligned with.

### Synthesis methods

Data was cleaned and summarised using SAS version 9.3. The constructs covered by each of the measures according to Moore et al's.  [7] definition of sustainability for measures assessing sustainability as an outcome, and the five higher-level domains from the Integrated Sustainability Framework [2] for measures of sustainability determinants, were summarised and organised in a table. Descriptive statistics were used to summarise the quality of each measure against the proposed nine psychometric indicators and five pragmatic domains outlined by PAPERS [17]. Where possible, a total quality rating score for each of the pragmatic and psychometric domains was calculated as well as overall, for each measure by summing together the relevant items. Total overall  scores range from a possible −14 to 56 [17, 31]. Summary tables were produced that included information describing the characteristics of the measure, the specific setting, and any sub-groups in which the measure has evidence of validity. The use of each measure in empirical studies was summarised descriptively.

## Results

### Search results

A total of 32,782 scientific articles were identified from the database search, from which 402 full texts were screened and 37 were included in the final review. An additional 186 relevant articles were identified from the grey literature search, resulting in 223 articles included in this review, representing 28 individual measures. See Additional file 2: Figure S1, for a summary of the article selection, and Additional file 2: Table S2 for a summary of exclusion reasons for measures included in previous reviews and repositories.

### Overview of identified measures

Table 1 describes the characteristics of the included measures. Two measures assessed sustainability as an outcome, 25 assessed sustainability determinants, and one explicitly assessed both. Four measures were designed to assess different constructs other than those more directly related to sustainability or sustainability determinants. Twenty measures were based on a theory or framework, and 20 (of the 28 measures) included input from the target population during the development stage.

Seventeen measures were developed or psychometrically evaluated in the USA, four in Australia, two in the Netherlands, and one each in Sweden and UK. Three measures were developed and/or psychometrically evaluated in more than one country. All 28 measures were available in English, while only five measures were also available in a language other than English.

In relation to the scope of the identified measures, 11 were general measures designed to assess sustainability as an outcome or sustainability determinants in relation to any type of EBI within any setting. Four were general in terms of the target EBI but were restricted to a particular setting (e.g., clinical, public health, school). Seven could be used within any setting but were designed for a specific EBI or category of EBIs (e.g., health promotion programs, community-based programs, chronic disease prevention programs). Three were designed for a specific type of EBI or category of EBIs within a specific setting (e.g., depression care within a clinical/health care setting). Three were developed for assessing determinants of sustainability for the same specific EBI, the school-wide positive behavioral interventions and supports programs, which is delivered within the school setting.

Twenty measures were designed to be completed by both executive (e.g., supervisors, directors, administrators) and frontline staff (i.e., staff responsible for the day-to-day delivery of the EBI). Three measures were designed to be completed by executive staff only, and two by frontline staff only. Three were completed by researchers or purveyors responsible for monitoring or supporting the implementation of an EBI.

### Content validity of identified measures

Table 2 describes the constructs covered by measures of sustainability according to Moore's definition [7]. All three measures that assessed sustainability as an outcome covered the continued delivery of the EBI, while both the Provider Report of Sustainment Scale (PRESS) measure and the sustainment sub-scale from the SMSS incorporated aspects of behavior change. Only one measure incorporated concepts of time, evolution/adaptation, and continued benefits. None of the three measures incorporated all five main concepts related to sustainability as an outcome.

**Table 2:** Constructs covered by measures of sustainability according to the multi-dimensional definition proposed by Moore et al. (2017) [7]

Measure	Time	Continued delivery	Behavior change	Evolution/adaptation	Continued benefits
Provider Report of Sustainment Scale (PRESS) [32]	No	Yes	Yes	Yes	No
Stages of implementation completion (SIC) [33–35]	Yes	Yes	No	No	No
Sustainment Measurement System Scale (SMSS) (Sustainment sub-scale)^a^ [72]	No	Yes	Yes	No	Yes

^a^Includes a specific sub-scale that assesses sustainability as an outcome and seven sub-scales assessing determinants of sustainability. Only the sustainability sub-scale is assessed here

Table 3 describes the constructs covered by the 26 measures of sustainability determinants according to the higher-order domains of the Integrated Sustainability Framework [2]. Ten measures covered aspects of all five higher-level domains. However, no measure covered all constructs that define the five higher-level, multi-level domains (see Additional file 2: Tables S3 to S7). “Inner context factors” was the most frequently covered domain with all but two measures (*n*=25) covering aspects of this domain. This was followed by the domains of “intervention characteristics” (*n*=23), “outer context” (*n*=18), “processes,” and “implementer and population characteristics” (*n*=17 measures each). When assessing the lower-level constructs that define the five higher-level domains of the Integrated Sustainability Framework, the “inner context factors” and “outer context factors” domains were the most broadly covered (Additional file 2: Tables S3 and S4). Conversely, the “interventionist and population” domain and “characteristics of the intervention” were the most sparsely covered domains with only one and no measures, respectively, assessing all aspects of these domains (Additional file 2: Table S6 and S7).

**Table 3:** Constructs covered by measures of sustainability determinants according to the domains of the Integrated Sustainability Framework by Shelton et al. (2018) [2]

Measure	Outer context	Inner context	Intervention characteristics	Processes	Implementation and population characteristics
Assessment of Barriers to Implementation and Sustainability in Schools (ABISS) [36]	No	Yes	No	No	Yes
Advanced Level Tier Interventions Treatment Utilization and Durability (ALTITUDE) [37]	Yes	Yes	Yes	Yes	No
A measurement instrument for sustainability of work practices in in long-term care – long version [38]	Yes	Yes	Yes	Yes	Yes
A measurement instrument for sustainability of work practices in in long-term care – short version [38]	Yes	Yes	Yes	Yes	Yes
Change Process Capability Questionnaire (CPCQ) [39]	No	Yes	Yes	Yes	Yes
Clinical Sustainability Assessment Tool (CSAT) [40]	Yes	Yes	Yes	Yes	Yes
Faith-Based Organization Health Integration Inventory (FBO-HII) [41]	Yes	Yes	Yes	No	No
General Organizational Index (GOI) [42, 43]	No	No	Yes	No	Yes
Levels of Institutionalisation (Loln) [44, 45]	No	Yes	Yes	Yes	Yes
National Health Service (NHS) Sustainability Model and Guide [47]	No	Yes	Yes	Yes	Yes
The Normalisation Measure Development questionnaire (NoMAD) [48–53]	No	Yes	Yes	Yes	Yes
New South Wales Sustainability Checklist [55]	Yes	Yes	Yes	No	Yes
Office of Adolescent Health (OAH) Sustainability Assessment [56]	Yes	Yes	Yes	No	Yes
Office of Population Affairs (OPA) Sustainability Assessment Tool [57]	Yes	Yes	Yes	No	No
Prevention Program Assessment [58]	Yes	Yes	Yes	Yes	Yes
Program Sustainability Assessment Tool (PSAT) [10]	Yes	Yes	Yes	Yes	No
Program Sustainability Assessment Tool (PSAT) – adapted for elementary setting [59]	Yes	Yes	Yes	Yes	No
Program Sustainability Index [60]	Yes	Yes	Yes	Yes	Yes
School-wide Universal Behaviour Sustainability Index- School Teams (SUBSIST) [61–65]	Yes	Yes	Yes	Yes	Yes
Sustainability Formative Self-Assessment Tool [66]	Yes	Yes	Yes	No	No
Sustainable Implementation Scale (SIS) [67]	Yes	Yes	Yes	No	No
Sustaining Innovation Through Education (SITE): Extended Behavioural [69]	Yes	Yes	Yes	Yes	Yes
Sustaining Innovation Through Education (SITE): Short Behavioural [69]	Yes	Yes	Yes	Yes	Yes
Sustained Implementation Support Scale [68]	No	Yes	No	Yes	No
Sustainment Leadership Scale [70]	No	Yes	No	No	No
Sustainment Measurement System Scale (SMSS)^a^ [72]	Yes	Yes	Yes	Yes	Yes

^a^Includes a sub-scale that assesses sustainability as an outcome and seven sub-scales assessing determinants of sustainability. Only the seven sub-scales assessing determinants of sustainability are assessed here

### Psychometric and pragmatic qualities of identified measures

Table 1 details the overall PAPERS score for each measure, which were calculated by summing the ratings obtained from the individual items assessing the psychometric qualities (Table 4) together with the ratings for the individual items assessing the pragmatic qualities (Table 5). The PRESS measure, which measures sustainability as an outcome, was the highest-rated measure overall, with a total score of 35. Of the measures of sustainability determinants, the School-wide Universal Behavior Sustainability Index - School Teams (SUBSIST) measure obtained the highest PAPERS score with 29, followed by the Clinical Sustainability Assessment Tool (CSAT) and Sustainment Measurement System Scale (SMSS) each with a score of 28. Specifically, the SUBSIST had a higher overall score due to a larger number of psychometric properties being assessed compared to the CSAT and SMSS.

**Table 4:** Psychometric ratings according to PAPERS* for identified measures of sustainability as an outcome and measures of sustainability determinants

Measure name	Internal consistency Median (range; ***n***)	Convergent validity Median (range; ***n***)	Discriminant validity Median (range; ***n***)	Known-groups validity Median (range; ***n***)	Predictive validity Median (range; ***n***)	Concurrent validity Median (range; ***n***)	Structural validity Median (range; ***n***)	Responsiveness Median (range; ***n***)	Norms Median (range; ***n***)	Total psychometric score
**Measures of sustainability as an outcome**										
Provider Report of Sustainment Scale (PRESS) [32]	4 (*n*=1)	4 (*n*=1)	0	2 (*n*=1)	0	0	4 (*n*=1)	0	4 (*n*=1)	**18**
Stages of implementation completion (SIC) – original [34]^a^	0	0	0	0	0	0	0	0	0	**0**
**Measures of sustainability determinants**										
Assessment of Barriers to Implementation and Sustainability in Schools (ABISS) [36]	2 (*n*=1)	0	0	3 (*n*=1)	0	1 (*n*=1)	2 (*n*=1)	0	2 (*n*=1)	**10**
Advanced Level Tier Interventions Treatment Utilization and Durability (ALTITUDE) [37]	4 (*n*=1)	0	0	0	0	1 (*n*=1)	2 (*n*=1)	0	0	**7**
A measurement instrument for sustainability of work practices in in long-term care – long version [38, 77]	3 (3, 4; *n*=2)	4 (*n*=1)	0	0	0	0	2 (*n*=1)	0	1 (*n*=1)	**10**
A measurement instrument for sustainability of work practices in in long-term care – short version [38]	3 (*n*=1)	0	0	0	0	0	2 (*n*=1)	0	1 (*n*=1)	**6**
Change Process Capability Questionnaire (CPCQ) [39]	4 (*n*=1)	0	0	0	0	1 (*n*=1)	0	0	−1 (*n*=1)	**4**
Clinical Sustainability Assessment Tool (CSAT) [40]	4 (*n*=1)	0	0	2 (*n*=1)	0	0	2 (*n*=1)	0	2 (*n*=1)	**10**
Faith-Based Organization Health Integration Inventory (FBO-HII) [41]	3 (*n*=1)	0	0	0	0	0	−1 (*n*=1)	0	0	**2**
General Organizational Index (GOI) [42, 43, 78]	2 (2, 2; *n*=2)	0	0	0	0	3 (2, 3; *n*=3)	0	4 (4,4; *n*=2)	−1 (−1, −1; *n*=2)	**8**
Levels of Institutionalization (Loln) [44, 45]	2 (1, 3; *n*=2)	3 (*n*=1)	0	1 (*n*=1)	0	1 (*n*=1)	1 (1, 2; *n*=2)	0	−1 (*n*=1)	**7**
National Health Service (NHS) Sustainability Model and Guide [47]	0	0	0	0	0	0	0	0	0	**0**
The Normalisation Measure Development questionnaire (NoMAD) [48, 49, 51]	2 (1, 3; *n*=2)	4 (*n*=1)	0	0	0	0	2 (n=1)	0	0	**8**
The Normalisation Measure Development questionnaire – Dutch (NoMAD) [50]	3 (*n*=1)	2 (*n*=1)	0	0	0	0	−1 (*n*=1)	0	2 (*n*=1)	**6**
The Normalisation Measure Development questionnaire - Portuguese (NoMAD) [52]	2 (*n*=1)	0	0	0	0	0	0	0	0	**2**
The Normalisation Measure Development questionnaire - Swedish (S-NoMAD) [53]	3 (*n*=1)	0	0	0	0	0	4 (*n*=1)	0	0	**7**
New South Wales Sustainability Checklist [55]	1 (*n*=1)	0	0	0	0	0	0	0	1 (*n*=1)	**2**
Office of Adolescent Health (OAH) Sustainability Assessment [56]	0	0	0	0	0	0	0	0	0	**0**
Office of Population Affairs (OPA) Sustainability Assessment Tool [57]	0	0	0	0	0	0	0	0	0	**0**
Prevention Program Assessment [58]	2 (*n*=1)	2 (*n*=1)	0	4 (*n*=1)	0	0	2 (*n*=1)	0	2 (*n*=1)	**12**
Program Sustainability Assessment Tool (PSAT) [10]	3 (*n*=1)	0	0	0	0	3 (*n*=1)	2 (*n*=1)	0	0	**8**
Program Sustainability Assessment Tool (PSAT) – adapted for elementary setting [59]	4 (*n*=1)	−1 (*n*=1)	0	0	0	0	−1 (*n*=1)	0	2 (*n*=1)	**4**
Program Sustainability Index [60]	2 (*n*=1)	2 (*n*=1)	0	0	0	0	2 (*n*=1)	0	2 (*n*=1)	**8**
School-wide Universal Behaviour Sustainability Index- School Teams (SUBSIST) [61–65]	4 (*n*=1)	3 ( *n*=1)	0	3 (2, 4; *n*=2)	−1 (*n*=1)	1 (1, 2; *n*=2)	2 (2, 3; *n*=2)	0	2 (−1, 4; *n*=3)	**14**
Sustainability Formative Self-Assessment Tool [66]	0	0	0	0	0	0	0	0	0	**0**
Sustainable Implementation Scale (SIS) [67]	4 (*n*=1)	0	0	2 (*n*=1)	0	4 (*n*=1)	0	0	−1 (*n*=1)	**9**
Sustained Implementation Support Scale [68]	4 (*n*=1)	0	0	0	0	1 (*n*=1)	2 (*n*=1)	0	0	**7**
Sustaining Innovation Through Education (SITE): Extended Behavioral [69]	4 (*n*=1)	0	0	0	0	0	0	0	0	**4**
Sustaining Innovation Through Education (SITE): Short Behavioral [69]	4 (*n*=1)	0	0	0	0	0	0	0	0	**4**
Sustainment Leadership Scale [70]	4 (*n*=1)	0	0	0	0	0	3 (*n*=1)	0	2 (*n*=1)	**9**
Sustainment Measurement System Scale (SMSS) [72]	3 (*n*=1)	2 (*n*=1)	4 (*n*=1)	0	0	0	2 (*n*=1)	0	2 (*n*=1)	**13**

*All individual psychometric PAPERS items are scored on a scale from −1 to 4, with higher scores representing a higher level of quality

^a^Only psychometric properties relating to the sustainability aspects of this scale were considered, and at the time of this review, none was found to be available for assessment. There are however properties relating to the other aspects of this scale [35, 79]

**Table 5:** Pragmatic ratings according to PAPERS* for identified measures of sustainability as an outcome and measures of sustainability determinants

Measure name	Cost Median	Reading Median	Training Median	Interpretation Median	Length Median (range; ***n***)	Total pragmatic score
**Measures of sustainability as an outcome**						
Provider Report of Sustainment Scale (PRESS) [32]	4	4	4	1	4	**17**
Stages of implementation completion (SIC) –original [33]	4	3	2	3	2 (2, 3, *n*=2)	**14**
**Measures of sustainability determinants**						
Assessment of Barriers to Implementation and Sustainability in Schools (ABISS) [36]	4	3	4	1	4	**16**
Advanced Level Tier Interventions Treatment Utilization and Durability (ALTITUDE) [37]	4	2	4	1	3	**14**
A measurement instrument for sustainability of work practices in in long-term care – long version [38]	4	4	4	1	3	**16**
A measurement instrument for sustainability of work practices in in long-term care – short version [38]	4	4	4	1	3	**16**
Change Process Capability Questionnaire (CPCQ) [39]	4	3	4	1	3	**15**
Clinical Sustainability Assessment Tool (CSAT) [40]	4	3	4	4	3	**18**
Faith-Based Organization Health Integration Inventory (FBO-HII) [41]	4	3	4	1	3	**15**
General Organizational Index (GOI) [42, 43]	4	3	3	3	3	**16**
Levels of Institutionalisation (Loln) [44, 45]	4	4	4	3	3	**18**
National Health Service (NHS) Sustainability Model and Guide [47]	4	3	3	3	4	**17**
The Normalisation Measure Development questionnaire (NoMAD) [48–53]	4	3	3	1	3	**14**
New South Wales Sustainability Checklist [55]	4	3	3	1	3	**14**
Office of Adolescent Health (OAH) Sustainability Assessment [56]	4	3	4	3	2	**16**
Office of Population Affairs (OPA) Sustainability Assessment Tool [57]	4	4	4	3	3	**18**
Prevention Program Assessment [58]	4	3	4	1	3	**15**
Program Sustainability Assessment Tool (PSAT) [10]	4	3	4	4	3	**18**
Program Sustainability Assessment Tool (PSAT) – adapted for elementary setting [59]	4	3	4	1	3	**15**
Program Sustainability Index [60]	4	3	4	1	3	**15**
School-wide Universal Behavior Sustainability Index - school teams (SUBSIST) [61–65]	4	3	4	1	3	**15**
Sustainability Formative Self-Assessment Tool [66]	4	2	3	3	3	**15**
Sustainable Implementation Scale (SIS) [67]	4	2	4	1	3	**14**
Sustained Implementation Support Scale [68]	4	4	4	1	3	**16**
Sustaining Innovation Through Education (SITE): extended behavioral [69]	4	3	3	2	2	**14**
Sustaining Innovation Through Education (SITE): short behavioral [69]	4	3	3	2	3	**15**
Sustainment Leadership Scale [70]	4	3	4	1	3	**15**
Sustainment Measurement System Scale (SMSS) [72]	4	3	4	1	3	**15**

^*^All individual pragmatic PAPERS items are scored on a scale from −1 to 4, with higher scores representing a higher level of quality

### Psychometric qualities

Table 4 details the median score for the psychometric quality indicators from the PAPERS scale for each measure. Overall, PRESS was rated the highest in psychometric quality with a score of 18 out of a possible 36, followed by the SUBSIST measure with a score of 14. At an individual psychometric property level, internal consistency was the most frequently assessed (84%, *n*=26), with median scores ranging from 1 (minimal/emerging) to 4 (excellent). The second most frequently assessed psychometric property was structural validity (61%, *n*=19; median range; −1 to 4); followed by norms (55%, *n*=17; median range: −1 to 4). Few measures were assessed for responsiveness (*n*=1) or predictive validity (*n*=1). Additional file 2: Figure S2 provides a head-to-head comparison of the psychometric ratings of included measures.

### Pragmatic qualities

Table 5 details the median scores for the pragmatic qualities assessed as part of the PAPERS rating scale for each measure. Overall, the Levels of Institutionalization (Loln), CSAT, OPA Sustainability Assessment Tool, and the Program Sustainability Assessment Tool (PSAT) were rated the highest in pragmatic quality, with each of these measures scoring 18 out of a possible 20. All three of these measures assessed determinants of sustainability. Of the three measures of sustainability as an outcome, the PRESS measure scored the highest with a total score of 17. All pragmatic items were scored for all measures, with most of the information obtained from grey literature sources, such as websites or publicly available scoring manuals. In terms of individual items, the cost was the most highly rated with all measures scoring excellent (score of 4), as they were freely available either publicly from a website, within a published manuscript, or accessed via contact with the authors. The most poorly scored pragmatic quality was “ease of interpretation,” with only two measures scoring the highest rating of excellent and 17 scoring minimal/emerging (score of 1). Additional file 2: Figure S3 provides a comparison of the pragmatic ratings of included measures.

### Empirical application of identified measures

Table 6 describes how each of the identified measures have been used in empirical research to date. Eleven measures have yet to be used in an empirical study; six of which were only published since 2020. The most frequently used measure of sustainability as an outcome was the Stages of Implementation Completion (SIC) measure, which has been used in 27 studies. For measures of determinants of sustainability, the most frequently used was the Change Process Capability Questionnaire (CPCQ) (*n*=34), followed by the Normalisation Measure Development questionnaire (NoMAD) (*n*=29) and Program Sustainability Assessment Tool (PSAT) (*n*=20). Geographically, the NoMAD was the most widely used across 15 countries. All other measures have been used in six  or fewer countries. Of the 16 measures that have been used in empirical research, six were used to assess constructs other than sustainability determinants or sustainability as an outcome. Eleven measures were adapted prior to their use, despite only two measures (SIC and NoMAD) having been explicitly designed for adaptation in primary research. The most common adaptations included: removing items, adding items, changing the wording of items, changing the response scale, and deleting domains.

**Table 6:** Empirical use of identified measures of sustainability as an outcome and measures of determinants of sustainability

Measures of sustainability as an outcome						
Measure (year of initial publication)	Number of studies	Country	Settings	Perspectives	Constructs other than sustainability	Adaptations made
Provider Report of Sustainment Scale (PRESS) (2021) [32]	0					
Stages of implementation completion (SIC) (2006) [33]	27	Australia Canada Denmark Mozambique Switzerland USA	Clinics Community Department of Veterans Affairs sites Juvenile justice Housing providers Primary care Public service systems Substance use disorder treatment agencies	Administrators/executives Champions Purveyor Providers Research staff	Implementation process/phases	*N*=14 Removed items Additional items Changed wording New domain Change response scale
Measures of sustainability determinants						
Assessment of Barriers to Implementation and Sustainability in Schools (ABISS) (2016) [36]	0					
Advanced Level Tier Interventions Treatment Utilization and Durability (ALTITUDE) (2021) [37]	0					
A measurement instrument for sustainability of work practices in in long-term care (2011)^a^ [38]	6	Netherlands USA	Community Hospital Long term care organisations	Executives Frontline staff	None	*N* = 6 Removed items Additional items Changed wording Deleted domains Changed response scale
Change Process Capability Questionnaire (CPCQ) (2008) [39]	34	Europe Japan Spain USA Vietnam	College Community Outpatient Substance Use Disorder Treatment Clinics Primary care Dental clinics/practices Radiology health care organizations	Executives Frontline staff	Capacity to change Change process Implementation Organizational capacity Readiness to change Readiness to manage change Quality improvement Adoption of new interventions	*N* = 19 Removed items Additional items Changed wording Deleted domains Other
Clinical Sustainability Assessment Tool (CSAT) (2021) [40]	5	Europe USA	Health care Hospital General practice	Executives Frontline staff	None	*N* = 0
Faith-Based Organization Health Integration Inventory (FBO-HII) (2020) [41]	1	USA	Churches	Frontline staff	None	N = 0
General Organizational Index (GOI) (2009) [42, 43]	9	Netherlands Norway USA	Community mental health Primary care Medical centre	Frontline staff Implementation monitors Research staff Trained raters	Implementation fidelity Operating characteristics of an organization Organizational change Penetration and general integration Quality of clinical care Quality improvement	*N* = 2 Additional items Changed wording Deleted domains Other
Levels of Institutionalization (Loln) (1993) [44, 45]	14	Australia Belgium Canada Uganda USA	Community Department of Veterans Affairs sites Health facilities Hospitals Public health and sports services department School	Executive Frontline staff	Institutionalisation	*N* = 7 Removed items Changed wording Deleted domains Changed response scale Other
National Health Service (NHS) Sustainability Model and Guide (2010) [47]	18	Canada South Africa Tanzania UK USA	Department of Veterans Affairs sites Hospital Primary care Patient homes University Medical center	Executive staff Frontline staff Implementation teams	None	*N* = 2 Deleted domains Changed response scale
The Normalisation Measure Development questionnaire(NoMAD) (2018) [48–53]	29	Albania Australia Brazil Canada Denmark France Germany Kosovo Netherlands Scotland Spain Sweden UK USA Wales	Community health General practice Health care service Hospital Mental health organizations Primary care Rural health services School University	Executive staff Frontline staff	Acceptance Adoption Contextual factors impacting the implementation Implementation process Perceptions of implementation Normalization Maintenance	*N* = 13 Removed items Additional items Changed wording New domains Deleted domains Changed response scale Other
New South Wales Sustainability Checklist (2009) [55]	1	Australia	School	Stakeholders/partners	None	*N* = 1 Removed items
Office of Adolescent Health (OAH) Sustainability Assessment (2014) [56]	0					
Office of Population Affairs (OPA) Sustainability Assessment Tool (2019) [57]	0					
Prevention Program Assessment (2012) [58]	2	USA	Community health clinics Clinical care and practice settings	Frontline staff	Readiness for evidence-based intervention	*N* = 0
Program Sustainability Assessment Tool (PSAT) (2014) [10]	20	Malaysia Philippines Puerto Rico Spain USA	Criminal courthouse/justice settings Fire department Government health departments and non-government or support groups with HIV advocacy Pharmacy Prenatal clinics Primary care Home settings School State and community health settings State agencies and coalitions Supportive housing agencies	Executives Frontline staff Research staff Stakeholders	None	*N* = 4 Additional items Changed wording Deleted domains Changed response scale
Program Sustainability Assessment Tool (PSAT) – adapted for elementary setting (2021) [59]	0					
Program Sustainability Index (2004) [60]	12	USA	Child welfare systems Community settings Department of Veterans Affairs sites Medical centers	Executives Frontline staff Stakeholders	None	*N* = 10 Removed items Additional items Changed wording Deleted domains Changed response scale Other
School-wide Universal Behaviour Sustainability Index- School Teams (SUBSIST) (2010) [61–65]	6	New Zealand USA	School	Executives Frontline staff	None	*N* = 2 Removed items Additional items Other
Sustainability Formative Self-Assessment Tool (2011) [66]	0					
Sustainable Implementation Scale (SIS) (2018) [67]	4	Sweden	Community mental health services Public employment services	Research staff	None	*N* = 0
Sustained Implementation Support Scale (2018) [67]	1	Australia	Aboriginal and Torres Strait Islander child welfare agencies	Frontline staff	None	*N* = 0
Sustaining Innovation Through Education (SITE): Extended Behavioural (2020) [69]	0					
Sustaining Innovation Through Education (SITE): Short Behavioural ( 2020) [69]	0					
Sustainment Leadership Scale (2018) [70]	0					
Sustainment Measurement System Scale (SMSS) (2020) [72]	0					

^a^Empirical use of the long and short version of this measure were assessed together

## Discussion

We identified a growing number of measures relating to sustainability determinants, and, to a lesser extent, measures of sustainability as an outcome. Despite this increase, we found that the included measures had limited coverage of the key constructs of sustainability and were of variable quality, and only a small number were consistently used in empirical studies. This review identifies areas where future research is warranted, to ensure improvements in this field while minimising research waste. It also provides important information that end-users can use to help compare and select the most appropriate measure for their setting.

### General considerations across all identified measures

Most of the measures identified were developed and/or psychometrically evaluated in the USA (20 out of 28), limiting their cross-cultural validity. This may also limit content coverage of constructs, as the outer context (related to broader policy and social context) has been identified as an important determinant of sustainability [2]. Only five of the 28 measures are available in languages other than English, of which only one, the NoMAD, has been translated and psychometrically evaluated in several languages. Translation and validation of measures is an extensive and costly process that requires specialised expertise [80]. This is a major limitation of the field and has implications for equity, as it highlights the inadequate access that non-English speaking populations and countries have to rigorous and standardised measures relating to sustainability. Without this access, researchers often create their own measures or alternatively, translate, and adapt existing measures without proper validation. Creating or leveraging existing research consortiums that share resources across groups may help avoid this.

Only 11 (two for sustainability as an outcome and nine for sustainability determinants) of the 28 identified measures were designed for general use (see Table 1). Fortunately, simple changes to the referent in a measure (e.g., changing the referenced EBI) should not alter the psychometric properties. In at least five [36, 37, 41, 59, 61] measures, the items appeared to have  content specific to the EBI and/or setting (beyond simple referent values) that would require extensive adaptation that may warrant new psychometric evidence. The advantages of generalised measures are the ability to standardise research, allowing for replication and comparability across studies, while reducing research waste due to use of one-off measures. The need for more generalised measures is emphasised by our finding that most measures were adapted before use in empirical studies in ways that might compromise their psychometric evidence. However, it can be difficult to ensure that generalised measures are sensitive and informative, as the issues affecting sustainability can vary and depend on the setting and EBI under investigation [2]. Item banks, informed by item response theory, strike a balance between generalisability and specificity of a measure. The resulting standardised measures include survey items tailored to specific characteristics, such as settings, populations, and/or EBIs, which have been calibrated to create standardised scores that are comparable across the tailored items [23]. The use of item banks for measures within implementation science is not a new concept and has been suggested by other reviews of implementation measures [23]. Despite such calls few efforts have launched to create item banks for implementation science, which may be a focus for research consortia in the future.

The majority of the included measures (*n*=20) were designed to be completed by both the executive/management staff, who oversee the implementation of an EBI, and frontline staff, responsible for the day-to-day delivery of an EBI (see Table 1). In most instances, both executive and frontline staff are required to report on all items, regardless of their role in EBI delivery. Only the SIC, Sustainable Implementation Scale (SIS) and SUBSIST scales seem to distinguish issues between these two roles with separate questions for the different types of staff. The issues impacting on sustainability exist at varying levels within organisations [2, 8, 59]. Therefore, different levels of staff roles may have limited understanding of some determinants of sustainability or aspects of sustainability. For example, frontline staff may not be aware of budgetary constraints that administrators manage. Conversely, management may not possess the same level of day-to-day EBI implementation knowledge as front-line staff. If participants cannot accurately respond to a measures item, the usefulness of the data collected is compromised. Different scales, or at least items, within a scale may need to be completed by different types of staff to ensure that the full range of issues impacting sustainability are accurately captured.

### Measures of sustainability as an outcome

Of the 28 included measures, only three were classified as measuring sustainability as an outcome. This may reflect the difficulties in adequately assessing sustainability as an outcome via self-report, standardised scales, to validly capture continued delivery and benefit of specific EBIs. Instead, it may be more appropriate to measure sustainability via other means, such as using a measure that asks directly about the continued delivery of the EBI or via observation. For instance, the SIC measure is an objective measure of the implementation process that records the timing and continued delivery of the main components of an EBI. It is also being extended to comprehensively cover the sustainability phase following implementation [81], as currently, it is focused predominantly on measuring the earlier phases of implementation. Following such extensions and their rigorous psychometric evaluation, the SIC will make for an appealing comprehensive measure of the implementation process, including the sustainability phase. However, in some instances (e.g., where resources and time may be limited), the SIC may not be appropriate as it is more complicated to administer, requiring specific training, input from multiple data sources, and completion by researchers and purveyors over an extended period of time. Alternatively, a general standardised measure such as the PRESS, which scored the highest of all measures on the PAPERS criteria, may be suitable in such instances where direct measurement of EBI delivery cannot be obtained. Importantly, despite its high relative rating, the PRESS still lacks evidence of important psychometric properties including predictive validity, concurrent validity, and responsiveness. Furthermore, none of the three measures of sustainability covered all five domains of Moore et al. [7] definition. This is likely due to most of the measure assessing more specific constructs or aspects of sustainability, rather than the broader definition of sustainability used by Moore. For instance, sustainment has been recognised as a distinct concept, defined as the ongoing delivery of an evidence-based intervention [2, 8, 11, 32] and which was the focus of some of the measures included in this review, including PRESS [32]. As we were attempting to provide a comprehensive review of all quantitative measures related to sustainability we took a broad definition and included any related measures to sustainability. When developing and selecting measures for use, it is essential that one clearly defines the target construct and selects a measure that clearly aligns with their construct of interest.

### Measures of determinants of sustainability

Compared to measures of sustainability as an outcome we identified a large number of measures that aligned with our definition of determinants of sustainability, with 26 (out of the total 28) measures identified. Eight of the 28 measures were published since 2020, highlighting a recent increase in measure development, but several limitations exist. In terms of content validity, only 10 covered all 5 higher-level domains of the Integrated Sustainability Framework (see Table 3). While some of the measures (e.g., Sustainment Leadership Scale) were designed to cover only specific domains of determinants, the trade-off is a lack of a comprehensive assessments of sustainability. Few measures comprehensively covered all aspects of the “outer contextual factors” domain, which is a critical domain warranting multiple perspectives.

In terms of the psychometric and pragmatic qualities, the quality of these measures varied substantially with the PAPERS ratings ranging from as low as 15 to as high as 29 out of a possible score of 56. For psychometric properties, the largest gaps relate to discriminant validity, predictive validity, and responsiveness, highlighting opportunities for future research. For the pragmatic criteria all measures rated well for the items of cost and language. However, ease of interpretation was rated as minimal/emerging for all but ten of the sustainability determinants measures (see Table 5). Very few provided explicit instructions on how to score and interpret the measure. In fact, only two measures, the “National Health Service (NHS) Sustainability Model and Guide” [47] and the “Office of Adolescent Health (OAH) Sustainability Assessment” [56] provided explicit and detailed cutoff values and labels to enable classification of those at a greater risk of not sustaining delivery of an EBI. However, neither of these two measures have undergone comprehensive psychometric evaluation, and thus, the validity of these cut-points has not yet been examined.

### Recommendations for use of current measures

Based on the evidence presented in this review, there are limitations to all identified measures of sustainability and determinants of sustainability. However, we recommend the following.

- If objective measures of sustainability are not available or feasible, the PRESS measure should be considered as a measure of sustainability as an outcome, as it is the most psychometrically robust and pragmatic to date. Future research should strive to establish evidence of predictive validity and responsiveness for the PRESS measure to further enhance its psychometric properties.
- For measures of determinants of sustainability SUBSIST had the highest PAPERS score of 29. If evaluating school-wide positive behavioral interventions and supports, the SUBSIST should be considered as a measure of sustainability determinants for this EBI. However, it is not appropriate when considering other EBIs.
- In the context of other EBIs the CSAT and SMSS both had an overall PAPERS rating of 28, illustrating favourable psychometric and pragmatic qualities compared to other measures of sustainability determinants. It is recommended that the CSAT is considered for use when assessing sustainability determinants in a clinical setting and SMSS for other settings.
- In general, researchers wishing to use measures to assess the determinants of sustainability should carefully assess the psychometric and pragmatic qualities of each measure, as well as the specific characteristics to which the measure was designed to assess. The information provided in the tables within this paper should assist end-users to select the most robust and suitable measure for their context.
- Furthermore, when selecting a measure for use, the specific construct wishing to be measured should be carefully considered and a measure selected that aligns with the construct of interest.

### Limitations

There are limitations that should be considered when interpreting these results. First, we only included measures that were explicitly stated to be designed for broad, standardised use. This decision was made to avoid inclusion of one-off study-specific measures. This process may have missed some relevant measures that could potentially be used elsewhere. Second, we only included quantitative measures as we were interested in reflective measures that offered an efficient and comprehensive means of measuring and tracking sustainability as an outcome and sustainability determinants. This decision resulted in the exclusion of several sustainability-related tools that can be used to help support the planning and assessment of sustainability (e.g., RE-AIM and extension of RE-AIM focused on sustainability [82, 83], Long-Term Success Tool [84]). While these tools are useful in planning for, or tracking aspects of sustainability, they are not designed solely for quantitative measurement and thus were beyond the scope of this review. These exclusions also highlight the difficulties that can be faced by researchers and practitioners when attempting to select an appropriate, rigorous, and standarised quantitative measure of these concepts. Third, we classified a measure as covering a particular construct of interest if it included at least one item relating to a construct. This is in contrast to other reviews that have used a criteria of at least two items [23, 76]. We used a more liberal approach to ensure that we did not underestimate the content coverage of current measure, as we were mostly interested in assessing whether measures were incorporating any aspect, even to a small extent, the specific constructs we were focused on. This may have overestimated the content validity of identified measures, as it is usually insufficient to adequately cover an entire construct with only one item. Four, we only searched the references lists of relevant reviews and not all eligible articles, which was a deviation from our original registered protocol. This deviation was due to the extensive volume of articles screened and identified. However, given the extensive search strategy employed, including published and grey literature, reference lists of previous reviews, snowball searching, and searching of online repositories of implementation measures, it is unlikely this deviation would have impacted significantly on our search results of eligible measures. Finally, we only evaluated the psychometric properties of measures using studies with data that was explicitly analyzed for psychometric evaluation. Studies with data analysed for other purposes and not with the aim of assessing the psychometric properties of the measure, for example, an empirical study assessing the association between the measure and another construct but not with the a-priori aim of assessing the measures validity, was not considered when scoring that measures’ psychometric properties. This approach was taken as it was considered to be the most appropriate as psychometric evaluations should be pre-specified, and was also the most manageable and conservative approach for a review of this size.

## Conclusion

This systematic review identified and evaluated the psychometric and pragmatic properties of standardised measures of sustainability as an outcome and sustainability determinants for use across community, public health, and clinical settings. It provides a comprehensive guide that researchers and stakeholders can use to select the most psychometrically robust, pragmatic, and relevant measure of sustainability and/or sustainability determinants available for their setting. It also highlights where future research is needed to improve the psychometric and pragmatic quality of the current measures in this field.

## Supplementary Information

**Additional file 1: **PRISMA checklist.

**Additional file 2: **Additional data extraction and results.

**Additional file 3: **Example data extraction fields.

## Data Availability

Data and materials relating to this review are available from the corresponding author on reasonable request.
