# Supplementary material for: Evaluation of measures of sustainability and sustainability determinants for use in community, public health, and clinical settings: a systematic review
Source: Implement Sci. 2022 Dec 13;17:81. doi: 10.1186/s13012-022-01252-1 (PMC9746194; doi:10.1186/s13012-022-01252-1)
Supplement: Supplementary file 2 — Additional file 2: Additional data extraction and results. [file 13012_2022_1252_MOESM2_ESM.docx]

**Additional file 1**

Table 1a. Search strategy used in databases Ovid MEDLINE(R) and Epub Ahead of Print, In-Process, In-Data-Review & Other Non-Indexed Citations and Daily

| **#** | **Searches** | **Results** |
| --- | --- | --- |
| 1 | sustain*.tw. | 378382 |
| 2 | implement*.tw. | 549579 |
| 3 | routini?ation.tw. | 232 |
| 4 | institutionali?ation.tw. | 5114 |
| 5 | adopt*.tw. | 273548 |
| 6 | 1 or 2 or 3 or 4 | 907272 |
| 7 | Reproducibility of Results/ | 416970 |
| 8 | valid*.tw. | 809003 |
| 9 | reliab*.tw. | 517629 |
| 10 | ceiling effect.tw. | 1950 |
| 11 | observer variation.tw. | 1119 |
| 12 | psychometric*.tw. | 50375 |
| 13 | program evaluation.tw. or program evaluation/ | 67237 |
| 14 | outcome assessment.tw. | 4206 |
| 15 | Quality Indicators, Health Care/og [Organization & Administration] | 867 |
| 16 | 7 or 8 or 9 or 10 or 11 or 12 or 13 or 14 or 15 | 1528423 |
| 17 | Quality Assurance, Health Care/og [Organization & Administration] | 8253 |
| 18 | evidence-based medicine/ | 74415 |
| 19 | public health/ | 85768 |
| 20 | health services/ or community health services/ | 57481 |
| 21 | medical service.tw. | 8376 |
| 22 | workplace/ | 24732 |
| 23 | "Delivery of Health Care"/mt, st, sn [Methods, Standards, Statistics & Numerical Data] | 21586 |
| 24 | health care/ | 96959 |
| 25 | Translational Medical Research/ | 11685 |
| 26 | "Quality of Health Care"/og [Organization & Administration] | 4455 |
| 27 | Quality improvement.tw. | 38737 |
| 28 | 18 or 19 or 20 or 21 or 22 or 23 or 24 or 25 or 26 or 27 | 382494 |
| **29** | **6 and 16 and 28** | **7222** |

Table 1b. Search strategy used in databases **Embase**

| **#** | **Searches** | **Results** |
| --- | --- | --- |
| 1 | sustain*.tw. | 505955 |
| 2 | implement*.tw. | 724104 |
| 3 | routini?ation.tw. | 275 |
| 4 | institutionali?ation.tw. | 7073 |
| 5 | adopt*.tw. | 349626 |
| 6 | 1 or 2 or 3 or 4 or 5 | 1505966 |
| 7 | reproducibility/ | 231396 |
| 8 | valid*.tw. | 1138550 |
| 9 | reliab*.tw. | 677640 |
| 10 | ceiling effect.tw. | 2759 |
| 11 | observer variation.tw. | 1686 |
| 12 | psychometric*.tw. | 62196 |
| 13 | program evaluation/ | 16457 |
| 14 | program evaluation.tw. | 4956 |
| 15 | outcome assessment.tw. | 5763 |
| 16 | (healthcare or health care) adj (quality indicator*).tw. | 81 |
| 17 | 7 or 8 or 9 or 10 or 11 or 12 or 13 or 14 or 15 or 16 | 1854368 |
| 18 | health care quality assurance*.tw. | 45 |
| 19 | healthcare quality assurance*.tw. | 23 |
| 20 | evidence based medicine/ | 115286 |
| 21 | public health/ | 206367 |
| 22 | health service/ | 171541 |
| 23 | community care/ | 61393 |
| 24 | medical service.tw. | 11016 |
| 25 | workplace/ | 45690 |
| 26 | health care delivery/ and (method* or standard* or statistic* or data).tw. | 57931 |
| 27 | health care/ | 121657 |
| 28 | translational research/ | 18932 |
| 29 | health care quality/ and (organi* or admin*).tw. | 34706 |
| 30 | Quality improvement.tw. | 61270 |
| 31 | 18 or 19 or 20 or 21 or 22 or 23 or 24 or 25 or 26 or 27 or 28 or 29 or 30 | 824525 |
| **32** | **6 and 17 and 31** | **11696** |

Table 1c. Search strategy used in databases **APA PsycInfo**

| **#** | **Searches** | **Results** |
| --- | --- | --- |
| 1 | sustain*.tw. | 82924 |
| 2 | implement*.tw. | 191283 |
| 3 | routini?ation.tw. | 383 |
| 4 | institutionali?ation.tw. | 5878 |
| 5 | adopt*.tw. | 100318 |
| 6 | 1 or 2 or 3 or 4 or 5 | 355326 |
| 7 | Reproducibility.tw. | 3197 |
| 8 | valid*.tw. | 268613 |
| 9 | reliab*.tw. | 162241 |
| 10 | ceiling effect.tw. | 963 |
| 11 | observer variation.tw. | 24 |
| 12 | psychometric*.tw. | 81566 |
| 13 | Program Evaluation/ | 13003 |
| 14 | program evaluation.tw. | 8946 |
| 15 | outcome assessment.tw. | 1456 |
| 16 | ((healthcare or health care) adj quality indicator*).tw. | 23 |
| 17 | 7 or 8 or 9 or 10 or 11 or 12 or 13 or 14 or 15 or 16 | 395433 |
| 18 | health care quality assurance*.tw. | 9 |
| 19 | healthcare quality assurance*.tw. | 8 |
| 20 | Evidence Based Practice/ | 18819 |
| 21 | Public Health/ | 23184 |
| 22 | health service*.tw. | 55849 |
| 23 | medical service*.tw. | 3768 |
| 24 | (workplace* or work place*).tw. | 41839 |
| 25 | Health Care Delivery/ | 21286 |
| 26 | health care.tw. | 117527 |
| 27 | Translational Medic*.tw. | 154 |
| 28 | "Quality of Care"/ | 13837 |
| 29 | Quality improvement*.tw. | 5729 |
| 30 | 18 or 19 or 20 or 21 or 22 or 23 or 24 or 25 or 26 or 27 or 28 or 29 | 254398 |
| **31** | **6 and 17 and 30** | **3957** |

Table 1d. Search strategy used in databases **CINAHL**

| **#** | **Query** | **Results** |
| --- | --- | --- |
| S1 | TI sustain* OR AB sustain* | 82,396 |
| S2 | TI implement* OR AB implement* | 205,739 |
| S3 | TI routini?ation OR AB routini?ation | 166 |
| S4 | TI institutionali?ation OR AB institutionali?ation | 2,567 |
| S5 | TI adopt* OR AB adopt* | 72,662 |
| S6 | S1 OR S2 OR S3 OR S4 OR S5 | 337,445 |
| S7 | (MH "Reproducibility of Results") | 65,543 |
| S8 | TI valid* OR AB valid* | 197,372 |
| S9 | TI reliab* OR AB reliab* | 116,939 |
| S10 | TI "ceiling effect" OR AB "ceiling effect" | 825 |
| S11 | TI "observer variation" OR AB "observer variation" | 178 |
| S12 | TI psychometric* OR AB psychometric* | 25,534 |
| S13 | (MH "Program Evaluation") | 43,527 |
| S14 | TI "program evaluation" OR AB "program evaluation" | 2,460 |
| S15 | TI "outcome assessment" OR AB "outcome assessment" | 1,734 |
| S16 | TI ( (healthcare or health care) n1 (quality indicator*) ) OR AB ( (healthcare or health care) n1 (quality indicator*) ) | 304 |
| S17 | S7 OR S8 OR S9 OR S10 OR S11 OR S12 OR S13 OR S14 OR S15 OR S16 | 355,559 |
| S18 | TI health care quality assurance* OR AB health care quality assurance* | 198 |
| S19 | TI "healthcare quality assurance*" OR AB "healthcare quality assurance*" | 7 |
| S20 | TI "evidence based" OR AB "evidence based" | 75,466 |
| S21 | (MH "Public Health") | 53,016 |
| S22 | (MH "Health Services") | 14,103 |
| S23 | (MH "Community Health Services") | 23,265 |
| S24 | TI "medical service" OR AB "medical service" | 2,665 |
| S25 | TI workplace OR AB workplace | 30,508 |
| S26 | (MH "Health Care Delivery/MT/ST") | 8,757 |
| S27 | TI ( "health care") OR AB ( "health care") | 217,256 |
| S28 | (MH "Translational Medical Research") | 67 |
| S29 | (MH "Quality of Health Care/AM") | 1,349 |
| S30 | (MH "Quality Improvement") | 58,972 |
| S31 | S18 OR S19 OR S20 OR S21 OR S22 OR S23 OR S24 OR S25 OR S26 OR S27 OR S28 OR S29 OR S30 | 446,303 |
| **S32** | **S6 AND S17 AND S31** | **9,273** |

Table 1E. Search strategy used in databases **Cochrane Library**

| **ID** | **Search** | **Hits** |
| --- | --- | --- |
| #1 | sustain*:ti,ab | 41802 |
| #2 | implement*:ti,ab | 42889 |
| #3 | routini?ation:ti,ab | 13 |
| #4 | institutionali?ation:ti,ab | 612 |
| #5 | adopt*:ti,ab | 13780 |
| #6 | (1-#5) | 92981 |
| #7 | MeSH descriptor: [Reproducibility of Results] this term only | 10872 |
| #8 | valid*:ti,ab | 54078 |
| #9 | reliab*:ti,ab | 25160 |
| #10 | ceiling effect:ti,ab | 622 |
| #11 | observer variation:ti,ab | 406 |
| #12 | psychometric*:ti,ab | 3870 |
| #13 | program evaluation:ti,ab | 16725 |
| #14 | MeSH descriptor: [Program Evaluation] this term only | 6253 |
| #15 | outcome assessment:ti,ab | 65737 |
| #16 | health care quality indicator*:ti,ab | 1326 |
| #17 | (2-#16) | 158879 |
| #18 | health care quality assurance:ti,ab | 394 |
| #19 | MeSH descriptor: [Evidence-Based Medicine] this term only | 905 |
| #20 | MeSH descriptor: [Public Health] this term only | 254 |
| #21 | MeSH descriptor: [Health Services] this term only | 454 |
| #22 | MeSH descriptor: [Community Health Services] this term only | 1053 |
| #23 | medical service:ti,ab | 6727 |
| #24 | MeSH descriptor: [Workplace] this term only | 866 |
| #25 | MeSH descriptor: [Delivery of Health Care] this term only | 785 |
| #26 | health care:ti,ab | 88510 |
| #27 | MeSH descriptor: [Translational Medical Research] this term only | 121 |
| #28 | MeSH descriptor: [Quality of Health Care] this term only | 888 |
| #29 | Quality improvement:ti,ab | 34979 |
| #30 | (3-#29) | 122660 |
| #31 | **(4, #17, #29)** | **1430** |

Table 1F. Search strategy used in databases **ERIC**

| Search strategy |
| --- |
| noft((sustain* or implement* or routini?ation or institutionali?ation or adopt*)) AND noft((“reproducibility or result*” or valid* or reliab* or “ceiling effect” or “observer variation” or psychometric* or “program evaluation” or “outcome assessment” or “health care quality indicator*” or “healthcare quality indicator*”)) AND noft((“evidence based medicine” or “public health” or “health service*” or “medical service*” or workplace or “translational medical research” or “health care” or “healthcare” or “Quality improvement”)) |

Table 1G. Search strategy used in databases **SCOPUS**

| Search strategy |
| --- |
| ( TITLE-ABS ( ( sustain* OR implement* OR routini?ation OR institutionali?ation OR adopt* ) ) AND TITLE-ABS ( ( "reproducibility of result*" OR valid* OR reliab* OR "ceiling effect" OR "observer variation" OR psychometric* OR "program evaluation" OR "outcome assessment" OR "health care quality indicator*" OR "healthcare quality indicator*" ) ) AND TITLE-ABS ( ( "evidence based medicine" OR "public health" OR "health service*" OR "medical service*" OR workplace OR "translational medical research" OR "health care" OR "healthcare" OR "Quality improvement" ) ) ) AND ( LIMIT-TO ( EXACTKEYWORD , "Human" ) OR LIMIT-TO ( EXACTKEYWORD , "Humans" ) ) AND ( LIMIT-TO ( DOCTYPE , "ar" ) OR LIMIT-TO ( DOCTYPE , "re" ) ) AND ( LIMIT-TO ( LANGUAGE , "English" ) ) |


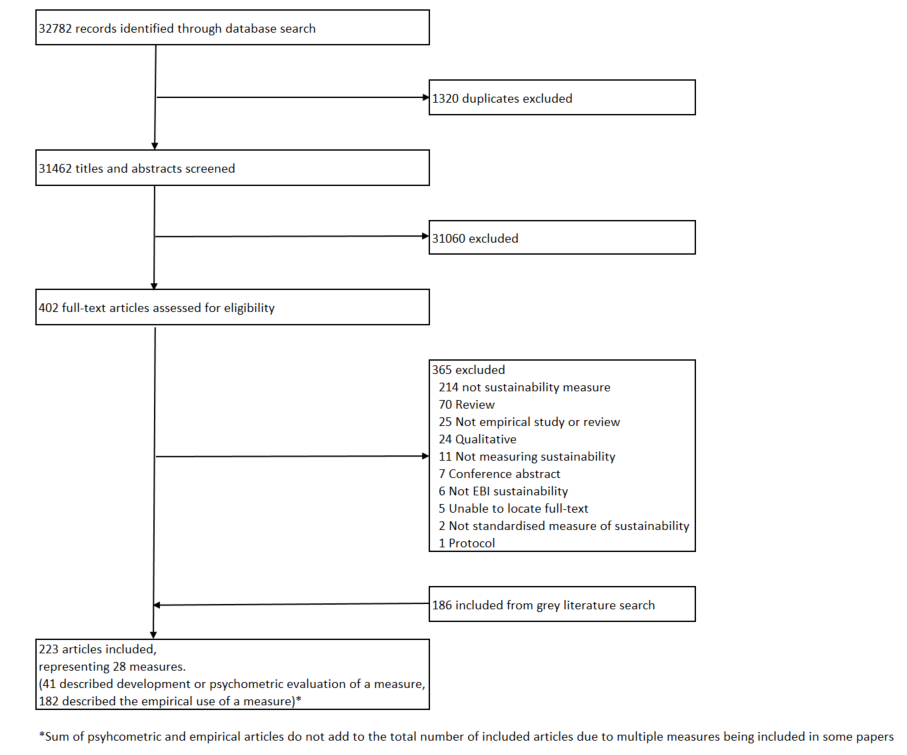


**Figure 1. Flow diagram illustrating measure selection**

**Table 2. Excluded measures or tools included in previous reviews or measure repositories**

| **Measure name** | **Author (year)** | **Review (year)** | **Reason for exclusion** |
| --- | --- | --- | --- |
| Amodeo Counselor Maintenance Measure | Amodeo (2010)(1) | Mettert (2020)(5)  Society for Implementation Research and Collaboration (SIRC)(6) | Not explicitly designed as a standardised measure of sustainability or sustainability determinants for broad use, designed for a specific study. |
| CBPR Model- Sustainability Measure | Sandoval (2012)(4)  Wallerstein (2008)(7)  Wallerstein (2010)(8) | Grid Enabled Measures Database (GEM)(9) | Limited information available on this measure. References listed in the GEM database do not provide information on the measure. The measure included on the GEM database not enough to assess. Does not seem to be a standardised measure for broad use. |
| COMMIT Questionnaire | Thompson (2000)(2) | GEM database(9) | Not explicitly designed as a standardised measure of sustainability or sustainability determinants for broad use, designed for a specific study. This was a survey developed for the aims of an individual study, which was to assess the sustainability of a tobacco control project. |
| Eisen Provider Knowledge & Attitudes Survey (Sustainability) | Eisen (2013)(10) | Mettert (2020)(5)  SIRC(6) | Not explicitly designed as a standardised measure of sustainability or sustainability determinants for broad use, designed for a specific study. This was a survey developed for the aims of an individual study, which was to assess the sustainability of the CATCH-IT program. |
| Evidence Based Practice Sustaining Telephone Survey | Swain (2009)(11) | Lewis (2015)(12);  Mettert (2020)(5);  Moullin (2020)(13)  SIRC(6) | Not explicitly designed as a standardised measure of sustainment or sustainability determinants for broad use, designed for a specific study. This was a survey developed for the aims of an individual study. Not a quantitative measure, includes both qualitative and quantitative questions. |
| Evidence-Based Organizational Checklist | Dorsey (2017)(14) | Lewis (2015)(12) | Not a measure of sustainability or sustainability determinants. This measure was designed to assess implementation climate, while this can relate to and support sustainment this was not a specific measure of a sustainability determinant. |
| Hospital Elder Life Program Sustainability Measure | Bradley (2005)(15) | GEM database(9) | Not explicitly designed as a standardised measure of sustainability or sustainability determinants for broad use, designed for a specific study. This was a survey developed for the aims of an individual study. Not a quantitative measure, but an open-ended discussion guide with broad questions. |
| Knowledge Exchange Outcomes Tool | Skinner (2007)(16) | Lewis (2015)(12);  Mettert (2020)(5)  SIRC(6) | Not a measure of sustainability or sustainability determinants. This measure was designed to assess reach and uptake of disseminated innovations. |
| Long Term Success Tool |  | Crespo-Gonzalez (2020)(17) | A tool for assessing and reporting individual items. No domain or overall score computed. |
| Not reported | Hodge (2016)(18) | Crespo-Gonzalez (2020)(17) | Not a specific measure of sustainability or sustainability determinants. It describes a review and conceptual model. |
| Not reported | Sridharan (2007)(19) | Crespo-Gonzalez (2020)(17) | Not a specific measure of sustainability or sustainability determinants. A set of items designed to measure whether strategic plans considered sustainment of a specific EBI. |
| Not reported | Amazigo (2007)(20) | Crespo-Gonzalez (2020)(17) | Not a specific measure of sustainability or sustainability determinants. It is a model protocol that collects data on indicators of sustainability for a specific project, with separate instruments developed to measure each indicator. The use of indicators suggests a formative rather than reflective model. |
| Not reported | Edwards (2007)(21) | Crespo-Gonzalez (2020)(17) | Not a specific measure of sustainability or sustainability determinants. Development of a framework and checklist designed to help with planning for sustainability. |
| Not reported | King (2018)(22) | Crespo-Gonzalez (2020)(17) | Not explicitly designed as a standardised measure of sustainability or sustainability determinants for broad use, designed for a specific study. This was a survey developed for the aims of an individual study to assess barriers and enablers to implementation. |
| Not reported | Ibrahim (2018)(23) | Crespo-Gonzalez (2020)(17) | Not explicitly designed as a standardised measure of sustainability or sustainability determinants for broad use, designed for a specific study. Semi-structured questionnaire developed for the aims of an individual study. |
| Not reported | Cooper (2015)(24) | Crespo-Gonzalez (2020)(17) | Not explicitly designed as a standardised measure of sustainability or sustainability determinants for broad use, designed for a specific study. This was a survey developed for the aims of an individual study to assess predictors of sustainability. |
| Not reported | Savaya (2009)(25) | Crespo-Gonzalez (2020)(17) | Not explicitly designed as a standardised measure of sustainability or sustainability determinants for broad use, designed for a specific study. This was a survey developed for the aims of an individual study to assess predictors of sustainability |
| Not reported | Hanh (2009)(26) | Crespo-Gonzalez (2020)(17) | Not a specific measure of sustainability or sustainability determinants. It is a framework with proposed indicators to sustainability. The use of indicators suggests a formative rather than reflective model. |
| Not reported | Feinberg (2008)(27)  Feinberg (2008b)(28) | Crespo-Gonzalez (2020)(17) | Not a specific measure of sustainability or sustainability determinants. Measure for planning for sustainability and a scale to assess board functioning. |
| Not reported | O’Loughlin (1998)(29) | Crespo-Gonzalez (2020)(17) | Not explicitly designed as a standardised measure of sustainability or sustainability determinants for broad use, designed for a specific study. This was a survey developed for the aims of an individual study to assess predictors of sustainability |
| Organization checklist |  | SIRC(6) | Classed as a measure of Evidence Based Practice Use. Not published and no indication if it is scored or not. |
| RE-AIM Planning Tool – Maintenance Measure |  | GEM database(9)  Moullin (2020)(13) | Not a specific measure of sustainability or sustainability determinants. It is a tool designed to help apply the RE-AIM domains to their project. It includes open-ended questions, thus not solely quantitative. |
| School Success Profile—Learning Organisation (SSP-LO) Measure | Bowen (2006)(30) | Clinton-McHarg (2016)(31) | Not a measure of sustainability or sustainability determinants. This measure was designed to assess organizational learning, while this can relate to and support sustainment this was not a specific measure of a sustainability determinant. |
| Sustainability and Spread Activities Questionnaire | Chung (2013)(32) | Mettert (2020)(5)  SIRC(6) | Not explicitly designed as a standardised measure of sustainability or sustainability determinants for broad use, designed for a specific study. This was a survey developed for the aims of an individual study. |
| Sustainability Dashboard | Sarriot (2009)(33) | Crespo-Gonzalez (2020)(17) | Not a specific measure of sustainability or sustainability determinants. It is a framework with proposed indicators to sustainability. The use of indicators suggests a formative rather than reflective model. |
| Sustainment Cost Survey | Roundfield (2017)(34) | Mettert (2020)(5)  SIRC(6) | Not a specific measure of sustainability or sustainability determinants. It describes a method for quantifying cost in implementation studies. |
| The Team Check-Up Tool (TCT) | Lubomski (2008)(35) | Moullin (2020)(13) | Not explicitly designed as a standardised measure of sustainability or sustainability determinants. Designed as a measure of identifying team activities and problems for goal achievements in relation to quality improvement activities. Also, more related to a planning and monitoring tool rather than a quantitative measure designed to measure an underlying construct. |
| Use of the RE-AIM Model Dimensions Items Checklist |  | Moullin (2020)(13) | Not a specific measure of sustainability or sustainability determinants. It is a tool designed to help evaluate the use of the RE-AIM domains in their project, rather than a specific quantitative measure. |

**Table 3. Lower-level constructs covered by measures of sustainability determinants for the higher-order domain outer contextual factors**

| **Measure** | **Socio-political context** | **Funding environment and availability** | **External partnerships and leadership/environmental support** | **Values, needs and priorities** |
| --- | --- | --- | --- | --- |
| Assessment of Barriers to Implementation and Sustainability in Schools (ABISS)(36) | No | No | No | No |
| Advanced Level Tier Interventions Treatment Utilization and Durability (ALTITUDE)(37) | No | No | Yes | No |
| A measurement instrument for sustainability of work practices in in long-term care – Long version(38) | No | No | Yes | No |
| A measurement instrument for sustainability of work practices in in long-term care – Short version(38) | No | No | Yes | No |
| Change Process Capability Questionnaire (CPCQ)(39) | No | No | No | No |
| Clinical Sustainability Assessment Tool (CSAT)(40) | No | No | Yes | Yes |
| Faith-Based Organization Health Integration Inventory (FBO-HII)(41) | No | No | Yes | Yes |
| General Organizational Index (GOI)(42, 43) | No | No | No | No |
| Levels of Institutionalisation (Loln)(44, 45) | No | No | No | No |
| National Health Service (NHS) Sustainability Model and Guide(46) | No | No | No | No |
| The Normalisation Measure Development questionnaire (NoMAD)(47-52) | No | No | No | No |
| New South Wales Sustainability Checklist(53) | No | Yes | Yes | Yes |
| OAH Sustainability Assessment(54) | Yes | Yes | Yes | Yes |
| OPA Sustainability Assessment Tool(55) | Yes | Yes | Yes | Yes |
| Prevention Program Assessment(56) | No | Yes | Yes | Yes |
| Program Sustainability Assessment Tool (PSAT)(57) | No | Yes | Yes | No |
| Program Sustainability Assessment Tool (PSAT) – adapted for elementary setting(58) | No | Yes | Yes | No |
| Program Sustainability Index(59) | No | Yes | Yes | Yes |
| School-wide Universal Behaviour Sustainability Index- School Teams (SUBSIST)(60-64) | Yes | Yes | Yes | Yes |
| Sustainability Formative Self-Assessment Tool(65) | No | No | Yes | Yes |
| Sustainable Implementation Scale (SIS)(66) | No | Yes | Yes | Yes |
| Sustained Implementation Support Scale(67) | No | No | No | No |
| Sustaining Innovation Through Education (SITE): Extended Behavioural(68) | Yes | Yes | Yes | Yes |
| Sustaining Innovation Through Education (SITE): Short Behavioural(68) | Yes | Yes | Yes | Yes |
| Sustainment Leadership Scale(69) | No | No | No | No |
| Sustainment Measurement System Scale (SMSS)(70) | No | Yes | Yes | No |

**Table 4. Lower-level constructs covered by measures of sustainability determinants for the higher-order domain inner contextual factors**

| **Measure** | **Programme champions** | **Organisational leadership/support** | **Organisational readiness/resources** | **Organisational stability** |
| --- | --- | --- | --- | --- |
| Assessment of Barriers to Implementation and Sustainability in Schools (ABISS)(36) | No | No | Yes | Yes |
| Advanced Level Tier Interventions Treatment Utilization and Durability (ALTITUDE)(37) | No | Yes | Yes | No |
| A measurement instrument for sustainability of work practices in in long-term care – Long version(38) | No | No | Yes | Yes |
| A measurement instrument for sustainability of work practices in in long-term care – Short version(38) | No | No | Yes | Yes |
| Change Process Capability Questionnaire (CPCQ)(39) | Yes | Yes | Yes | No |
| Clinical Sustainability Assessment Tool (CSAT)(40) | Yes | Yes | Yes | No |
| Faith-Based Organization Health Integration Inventory (FBO-HII)(41) | No | No | Yes | Yes |
| General Organizational Index (GOI)(42, 43) | No | No | No | No |
| Levels of Institutionalisation (Loln)(44, 45) | No | Yes | Yes | Yes |
| National Health Service (NHS) Sustainability Model and Guide(46) | No | Yes | No | No |
| The Normalisation Measure Development questionnaire (NoMAD)(47-52) | Yes | Yes | Yes | No |
| New South Wales Sustainability Checklist(53) | Yes | Yes | Yes | Yes |
| OAH Sustainability Assessment(54) | Yes | Yes | Yes | No |
| OPA Sustainability Assessment Tool(55) | Yes | Yes | Yes | Yes |
| Prevention Program Assessment(56) | No | Yes | Yes | Yes |
| Program Sustainability Assessment Tool (PSAT)(57) | Yes | Yes | Yes | Yes |
| Program Sustainability Assessment Tool (PSAT) – adapted for elementary setting(58) | Yes | Yes | Yes | Yes |
| Program Sustainability Index(59) | No | Yes | Yes | Yes |
| School-wide Universal Behaviour Sustainability Index- School Teams (SUBSIST)(60-64) | No | Yes | Yes | Yes |
| Sustainability Formative Self-Assessment Tool(65) | No | Yes | No | No |
| Sustainable Implementation Scale (SIS)(66) | Yes | Yes | Yes | Yes |
| Sustained Implementation Support Scale(67) | No | Yes | Yes | No |
| Sustaining Innovation Through Education (SITE): Extended Behavioural(68) | No | Yes | Yes | No |
| Sustaining Innovation Through Education (SITE): Short Behavioural(68) | No | Yes | Yes | No |
| Sustainment Leadership Scale(69) | No | Yes | No | No |
| Sustainment Measurement System Scale (SMSS)(70) | Yes | No | Yes | Yes |

**Table 5. Lower-level constructs covered by measures of sustainability determinants for the higher-order domain processes**

| **Measure** | **Partnership/ engagement** | **Training/supervision /support** | **Programme evaluation/data** | **Adaptation** | **Communications and strategic planning** |
| --- | --- | --- | --- | --- | --- |
| Assessment of Barriers to Implementation and Sustainability in Schools (ABISS)(36) | No | No | No | No | No |
| Advanced Level Tier Interventions Treatment Utilization and Durability (ALTITUDE)(37) | Yes | Yes | Yes | Yes | Yes |
| A measurement instrument for sustainability of work practices in in long-term care – Long version(38) | No | Yes | Yes | Yes | Yes |
| A measurement instrument for sustainability of work practices in in long-term care – Short version(38) | No | Yes | Yes | Yes | Yes |
| Change Process Capability Questionnaire (CPCQ)(39) | No | No | No | Yes | No |
| Clinical Sustainability Assessment Tool (CSAT)(40) | Yes | Yes | Yes | No | No |
| Faith-Based Organization Health Integration Inventory (FBO-HII)(41) | No | Yes | Yes | Yes | Yes |
| General Organizational Index (GOI)(42, 43) | No | Yes | Yes | No | No |
| Levels of Institutionalisation (Loln)(44, 45) | No | Yes | Yes | No | Yes |
| National Health Service (NHS) Sustainability Model and Guide(46) | No | Yes | Yes | No | Yes |
| The Normalisation Measure Development questionnaire (NoMAD)(47-52) | No | Yes | Yes | No | No |
| New South Wales Sustainability Checklist(53) | No | Yes | No | No | No |
| OAH Sustainability Assessment(54) | Yes | Yes | Yes | Yes | Yes |
| OPA Sustainability Assessment Tool(55) | Yes | No | Yes | No | Yes |
| Prevention Program Assessment(56) | Yes | Yes | Yes | No | Yes |
| Program Sustainability Assessment Tool (PSAT)(57) | Yes | No | Yes | Yes | Yes |
| Program Sustainability Assessment Tool (PSAT) – adapted for elementary setting(58) | Yes | Yes | Yes | Yes | Yes |
| Program Sustainability Index(59) | Yes | No | Yes | Yes | Yes |
| School-wide Universal Behaviour Sustainability Index- School Teams (SUBSIST)(60-64) | Yes | Yes | Yes | Yes | Yes |
| Sustainability Formative Self-Assessment Tool(65) | No | Yes | Yes | No | Yes |
| Sustainable Implementation Scale (SIS)(66) | No | Yes | Yes | No | Yes |
| Sustained Implementation Support Scale(67) | No | No | No | No | No |
| Sustaining Innovation Through Education (SITE): Extended Behavioural(68) | No | Yes | Yes | Yes | Yes |
| Sustaining Innovation Through Education (SITE): Short Behavioural(68) | No | Yes | No | No | Yes |
| Sustainment Leadership Scale(69) | No | No | No | No | No |
| Sustainment Measurement System Scale (SMSS)(70) | Yes | Yes | Yes | No | Yes |

**Table 6. Lower-level constructs covered by measures of sustainability determinants for the higher-order domain interventionists and population**

| **Measure** | **Implementer characteristics** | **Implementer benefits and stressors** | **Implementer skills/expertise** | **Population characteristics** |
| --- | --- | --- | --- | --- |
| Assessment of Barriers to Implementation and Sustainability in Schools (ABISS)(36) | No | No | No | No |
| Advanced Level Tier Interventions Treatment Utilization and Durability (ALTITUDE)(37) | Yes | No | Yes | No |
| A measurement instrument for sustainability of work practices in in long-term care – Long version(38) | Yes | No | Yes | No |
| A measurement instrument for sustainability of work practices in in long-term care – Short version(38) | Yes | No | Yes | No |
| Change Process Capability Questionnaire (CPCQ)(39) | Yes | Yes | No | No |
| Clinical Sustainability Assessment Tool (CSAT)(40) | Yes | No | No | No |
| Faith-Based Organization Health Integration Inventory (FBO-HII)(41) | No | No | No | No |
| General Organizational Index (GOI)(42, 43) | No | No | No | No |
| Levels of Institutionalisation (Loln)(44, 45) | Yes | No | No | No |
| National Health Service (NHS) Sustainability Model and Guide(46) | Yes | No | Yes | No |
| The Normalisation Measure Development questionnaire (NoMAD)(47-52) | Yes | Yes | Yes | No |
| New South Wales Sustainability Checklist(53) | No | No | No | No |
| OAH Sustainability Assessment(54) | No | No | No | No |
| OPA Sustainability Assessment Tool(55) | No | No | No | No |
| Prevention Program Assessment(56) | No | No | Yes | No |
| Program Sustainability Assessment Tool (PSAT)(57) | No | No | No | No |
| Program Sustainability Assessment Tool (PSAT) – adapted for elementary setting(58) | No | No | No | No |
| Program Sustainability Index(59) | Yes | No | Yes | Yes |
| School-wide Universal Behaviour Sustainability Index- School Teams (SUBSIST)(60-64) | Yes | Yes | Yes | Yes |
| Sustainability Formative Self-Assessment Tool(65) | No | No | No | No |
| Sustainable Implementation Scale (SIS)(66) | No | No | No | No |
| Sustained Implementation Support Scale(67) | No | Yes | No | No |
| Sustaining Innovation Through Education (SITE): Extended Behavioural(68) | Yes | Yes | Yes | No |
| Sustaining Innovation Through Education (SITE): Short Behavioural(68) | Yes | Yes | Yes | No |
| Sustainment Leadership Scale(69) | No | No | No | No |
| Sustainment Measurement System Scale (SMSS)(70) | Yes | No | Yes | No |

**Table 7. Lower-level constructs covered by measures of sustainability determinants for the higher-order domain characteristics of the intervention**

| **Measure** | **Adaptability of EBI/fidelity** | **Fit with context/population/ organisation** | **Perceived benefits** | **Perceived need** |
| --- | --- | --- | --- | --- |
| Assessment of Barriers to Implementation and Sustainability in Schools (ABISS)(36) | No | No | Yes | No |
| Advanced Level Tier Interventions Treatment Utilization and Durability (ALTITUDE)(37) | No | No | No | No |
| A measurement instrument for sustainability of work practices in in long-term care – Long version(38) | Yes | Yes | No | No |
| A measurement instrument for sustainability of work practices in in long-term care – Short version(38) | Yes | Yes | No | No |
| Change Process Capability Questionnaire (CPCQ)(39) | No | No | Yes | No |
| Clinical Sustainability Assessment Tool (CSAT)(40) | No | Yes | Yes | No |
| Faith-Based Organization Health Integration Inventory (FBO-HII)(41) | No | No | No | No |
| General Organizational Index (GOI)(42, 43) | Yes | No | No | No |
| Levels of Institutionalisation (Loln)(44, 45) | Yes | No | No | No |
| National Health Service (NHS) Sustainability Model and Guide(46) | Yes | Yes | Yes | No |
| The Normalisation Measure Development questionnaire (NoMAD)(47-52) | Yes | No | Yes | Yes |
| New South Wales Sustainability Checklist(53) | No | No | No | Yes |
| OAH Sustainability Assessment(54) | Yes | No | No | No |
| OPA Sustainability Assessment Tool(55) | No | No | No | No |
| Prevention Program Assessment(56) | Yes | No | No | Yes |
| Program Sustainability Assessment Tool (PSAT)(57) | Yes | No | Yes | No |
| Program Sustainability Assessment Tool (PSAT) – adapted for elementary setting(58) | Yes | No | Yes | No |
| Program Sustainability Index(59) | Yes | No | No | No |
| School-wide Universal Behaviour Sustainability Index- School Teams (SUBSIST)(60-64) | Yes | Yes | Yes | No |
| Sustainability Formative Self-Assessment Tool(65) | No | No | No | No |
| Sustainable Implementation Scale (SIS)(66) | No | No | No | No |
| Sustained Implementation Support Scale(67) | No | No | No | No |
| Sustaining Innovation Through Education (SITE): Extended Behavioural(68) | Yes | No | Yes | Yes |
| Sustaining Innovation Through Education (SITE): Short Behavioural(68) | No | No | Yes | Yes |
| Sustainment Leadership Scale(69) | No | No | No | No |
| Sustainment Measurement System Scale (SMSS)(70) | Yes | Yes | No | No |

**Figure 2. Psychometric ratings according to PAPERS for identified measures of sustainability as an outcome and measures of sustainability determinants**

**Figure 3. Pragmatic ratings according to PAPERS for identified measures of sustainability as an outcome and measures of sustainability determinants**

**References:**

1. Amodeo M, Storti SA, Larson MJ. Moving empirically-supported treatment to the workplace: recruiting addiction program supervisors to help in technology transfer. Substance use & misuse. 2010;45(6):968.

2. Thompson B, Lichtenstein E, Corbett K, Nettekoven L, Feng Z. Durability of tobacco control efforts in the 22 Community Intervention Trial for Smoking Cessation (COMMIT) communities 2 years after the end of intervention. Health Education Research. 2000;15(3):353-66.

3. Buhrmann L, Schuurmans J, Ruwaard J, Fleuren M, Etzelmuller A, Piera-Jimenez J, et al. Tailored implementation of internet-based cognitive behavioural therapy in the multinational context of the ImpleMentAll project: a study protocol for a stepped wedge cluster randomized trial. Trials. 2020;21(1):893.

4. Sandoval JA, Lucero J, Oetzel J, Avila M, Belone L, Mau M, et al. Process and outcome constructs for evaluating community-based participatory research projects: a matrix of existing measures. Health Education Research. 2012;27(4):680-90.

5. Mettert K, Lewis C, Dorsey C, Halko H, Weiner B. Measuring implementation outcomes: An updated systematic review of measures’ psychometric properties. Implementation Research and Practice. 2020;1.

6. Society for Implementation Research and Collaboration. Sustainability Instruments [Available from: h<ttps://societyforimplementationresearchcollaboration.org/sustainability-measures/.>

7. Wallerstein N, Oetzel J, Duran B, Tafoya G, Belone L, Rae R. What predicts outcomes in CBPR. Community-based participatory research for health: From process to outcomes. 2008;2:371-92.

8. Wallerstein N, Duran B. Community-based participatory research contributions to intervention research: the intersection of science and practice to improve health equity. American journal of public health. 2010;100(S1):S40-S6.

9. Grid-Enabled Measures Database. GEM [Available from: <https://www.gem-measures.org/Login.aspx?ReturnURL=Public/Measurelist.aspx?cat=2>.

10. Eisen JC, Marko-Holguin M, Fogel J, Cardenas A, Bahn M, Bradford N, et al. Pilot study of implementation of an internet-based depression prevention intervention (CATCH-IT) for adolescents in 12 US primary care practices: clinical and management/organizational behavioral perspectives. The Primary Care Companion for CNS Disorders. 2013;15(6):27106.

11. Swain K, Whitley R, McHugo GJ, Drake RE. The sustainability of evidence-based practices in routine mental health agencies. Community mental health journal. 2010;46(2):119-29.

12. Lewis CC, Fischer S, Weiner BJ, Stanick C, Kim M, Martinez RG. Outcomes for implementation science: an enhanced systematic review of instruments using evidence-based rating criteria. Implement Sci. 2015;10:155.

13. Moullin JC, Sklar M, Green A, Dickson KS, Stadnick NA, Reeder K, et al. Advancing the pragmatic measurement of sustainment: a narrative review of measures. Implement Sci Commun. 2020;1:76.

14. Dorsey S, Pullmann MD, Kerns SE, Jungbluth N, Meza R, Thompson K, et al. The juggling act of supervision in community mental health: Implications for supporting evidence-based treatment. Administration and Policy in Mental Health and Mental Health Services Research. 2017;44(6):838-52.

15. Bradley EH, Webster TR, Baker D, Schlesinger M, Inouye SK. After adoption: sustaining the innovation a case study of disseminating the hospital elder life program. Journal of the American Geriatrics Society. 2005;53(9):1455-61.

16. Skinner K. Developing a tool to measure knowledge exchange outcomes. The Canadian Journal of Program Evaluation. 2007;22(1):49.

17. Crespo-Gonzalez C, Benrimoj SI, Scerri M, Garcia-Cardenas V. Sustainability of innovations in healthcare: A systematic review and conceptual framework for professional pharmacy services. Research in Social and Administrative Pharmacy. 2020;16(10):1331-43.

18. Hodge LM, Turner KM. Sustained implementation of evidence‐based programs in disadvantaged communities: A conceptual framework of supporting factors. American journal of community psychology. 2016;58(1-2):192-210.

19. Sridharan S, Go S, Zinzow H, Gray A, Barrett MG. Analysis of strategic plans to assess planning for sustainability of comprehensive community initiatives. Evaluation and Program Planning. 2007;30(1):105-13.

20. Amazigo U, Okeibunor J, Matovu V, Zoure H, Bump J, Seketeli A. Performance of predictors: evaluating sustainability in community-directed treatment projects of the African programme for onchocerciasis control. Social science & medicine. 2007;64(10):2070-82.

21. Edwards JC, Feldman PH, Sangl J, Polakoff D, Stern G, Casey D. Sustainability of partnership projects: a conceptual framework and checklist. The Joint Commission Journal on Quality and Patient Safety. 2007;33(12):37-47.

22. King JC, Hibbs R, Saville CW, Swales MA. The survivability of dialectical behaviour therapy programmes: a mixed methods analysis of barriers and facilitators to implementation within UK healthcare settings. BMC psychiatry. 2018;18(1):1-11.

23. Ibrahim U, Wan-Puteh SE. An overview of civil society organizations’ roles in health project sustainability in Bauchi State, Nigeria. The Pan African Medical Journal. 2018;30.

24. Cooper BR, Bumbarger BK, Moore JE. Sustaining evidence-based prevention programs: Correlates in a large-scale dissemination initiative. Prevention Science. 2015;16(1):145-57.

25. Savaya R, Elsworth G, Rogers P. Projected sustainability of innovative social programs. Evaluation Review. 2009;33(2):189-205.

26. Hanh TTT, Hill PS, Kay BH, Quy TM. Development of a framework for evaluating the sustainability of community-based dengue control projects. The American journal of tropical medicine and hygiene. 2009;80(2):312-8.

27. Feinberg ME, Gomez BJ, Puddy RW, Greenberg MT. Evaluation and community prevention coalitions: Validation of an integrated web-based/technical assistance consultant model. Health Education & Behavior. 2008;35(1):9-21.

28. Feinberg ME, Bontempo DE, Greenberg MT. Predictors and level of sustainability of community prevention coalitions. American journal of preventive medicine. 2008;34(6):495-501.

29. O'Loughlin J, Renaud L, Richard L, Gomez LS, Paradis G. Correlates of the sustainability of community-based heart health promotion interventions. Preventive medicine. 1998;27(5):702-12.

30. Bowen GL, Rose RA, Ware WB. The reliability and validity of the school success profile learning organization measure. Evaluation and program planning. 2006;29(1):97-104.

31. Clinton-McHarg T, Yoong SL, Tzelepis F, Regan T, Fielding A, Skelton E, et al. Psychometric properties of implementation measures for public health and community settings and mapping of constructs against the Consolidated Framework for Implementation Research: a systematic review. Implement Sci. 2016;11(1):148.

32. Chung H, Duffy FF, Katzelnick DJ, Williams MD, Trivedi MH, Rae DS, et al. Sustaining practice change one year after completion of the national depression management leadership initiative. Psychiatric Services. 2013;64(7):703-6.

33. Sarriot E, Ricca J, Ryan L, Basnet J, Arscott‐Mills S. Measuring sustainability as a programming tool for health sector investments: report from a pilot sustainability assessment in five Nepalese health districts. The International journal of health planning and management. 2009;24(4):326-50.

34. Roundfield KD, Lang JM. Costs to community mental health agencies to sustain an evidence-based practice. Psychiatric services. 2017;68(9):876-82.

35. Lubomski LH, Marsteller JA, Hsu Y-J, Goeschel CA, Holzmueller CG, Pronovost PJ. The team checkup tool: evaluating QI team activities and giving feedback to senior leaders. Joint Commission Journal on Quality and Patient Safety. 2008;34(10):619-23.

36. Turri MG, Mercer SH, McIntosh K, Nese RN, Strickland-Cohen MK, Hoselton R. Examining barriers to sustained implementation of school-wide prevention practices. Assessment for Effective Intervention. 2016;42(1):6-17.

37. Kittelman A, Mercer SH, McIntosh K, Nese RN. Development and validation of a measure assessing sustainability of tier 2 and 3 behavior support systems. Journal of School Psychology. 2021;85:140-54.

38. Slaghuis SS, Strating M, Bal R, Nieboer AP. A measurement instrument for spread of quality improvement in healthcare. International Journal for Quality in Health Care. 2013;25(2):125-31.

39. Solberg LI, Asche SE, Margolis KL, Whitebird RR. Measuring an organization's ability to manage change: the change process capability questionnaire and its use for improving depression care. American Journal of Medical Quality. 2008;23(3):193-200.

40. Malone S, Prewitt K, Hackett R, Lin JC, McKay V, Walsh-Bailey C, et al. The Clinical Sustainability Assessment Tool: measuring organizational capacity to promote sustainability in healthcare. Implementation science communications. 2021;2(1):1-12.

41. Williams RM, Zhang J, Woodard N, Slade J, Santos SLZ, Knott CL. Development and validation of an instrument to assess institutionalization of health promotion in faith-based organizations. Evaluation and Program Planning. 2020;79:101781.

42. Bond GR, Drake RE, Rapp CA, McHugo GJ, Xie H. Individualization and quality improvement: Two new scales to complement measurement of program fidelity. Administration and Policy in Mental Health and Mental Health Services Research. 2009;36(5):349-57.

43. Heiervang KS, Egeland KM, Landers M, Ruud T, Joa I, Drake RE, et al. Psychometric properties of the general organizational index (GOI): a measure of individualization and quality improvement to complement program fidelity. Administration and Policy in Mental Health and Mental Health Services Research. 2020;47(6):920-6.

44. Barab SA, Redman BK, Froman RD. Measurement characteristics of the levels of institutionalization scales: examining reliability and validity. Journal of nursing measurement. 1998;6(1):19-33.

45. Goodman RM, McLeroy KR, Steckler AB, Hoyle RH. Development of level of institutionalization scales for health promotion programs. Health education quarterly. 1993;20(2):161-78.

46. Maher L GD, Evans A. . Sustainability Model and Guide. 2010.

47. Finch TL, Girling M, May CR, Mair FS, Murray E, Treweek S, et al. Improving the normalization of complex interventions: part 2-validation of the NoMAD instrument for assessing implementation work based on normalization process theory (NPT). BMC medical research methodology. 2018;18(1):1-13.

48. Rapley T, Girling M, Mair FS, Murray E, Treweek S, McColl E, et al. Improving the normalization of complex interventions: part 1-development of the NoMAD instrument for assessing implementation work based on normalization process theory (NPT). BMC Medical Research Methodology. 2018;18(1):1-17.

49. Vis C, Ruwaard J, Finch T, Rapley T, de Beurs D, van Stel H, et al. Toward an objective assessment of implementation processes for innovations in health care: psychometric evaluation of the normalization measure development (NoMAD) questionnaire among mental health care professionals. Journal of medical Internet research. 2019;21(2):e12376.

50. Davis S. Ready for Prime Time? Using Normalization Process Theory to Evaluate Implementation Success of Personal Health Records Designed for Decision Making. Frontiers in digital health. 2020:28.

51. Loch AP, Finch T, Fonsi M, Soárez PCd. Cross-cultural adaptation of the NoMAD questionnaire to Brazilian Portuguese. Revista da Associação Médica Brasileira. 2020;66:1383-90.

52. Elf M, Nordmark S, Lyhagen J, Lindberg I, Finch T, Åberg AC. The Swedish version of the Normalization Process Theory Measure S-NoMAD: translation, adaptation, and pilot testing. Implementation Science. 2018;13(1):1-12.

53. Hawe P KL, Noort M, Jordens C, Lloyd B. Indicators to help with capacity building in health promotion. 2000.

54. Office of Adolescent Health. Building sustainable programs: The resource guide. 2014.

55. Office of Population Affairs. Resource guide for building sustainable programs. 2019.

56. Stamatakis KA, McQueen A, Filler C, Boland E, Dreisinger M, Brownson RC, et al. Measurement properties of a novel survey to assess stages of organizational readiness for evidence-based interventions in community chronic disease prevention settings. Implementation Science. 2012;7(1):1-10.

57. Luke DA, Calhoun A, Robichaux CB, Elliott MB, Moreland-Russell S. Peer reviewed: The program sustainability assessment tool: A new instrument for public health programs. Preventing chronic disease. 2014;11.

58. Hall A, Shoesmith A, Shelton RC, Lane C, Wolfenden L, Nathan N. Adaptation and Validation of the Program Sustainability Assessment Tool (PSAT) for Use in the Elementary School Setting. International journal of environmental research and public health. 2021;18(21):11414.

59. Mancini JA, Marek LI. Sustaining community‐based programs for families: conceptualization and measurement. Family Relations. 2004;53(4):339-47.

60. Hume A, McIntosh K. Construct validation of a measure to assess sustainability of school‐wide behavior interventions. Psychology in the Schools. 2013;50(10):1003-14.

61. Kittelman A, Bromley KW, Mercer SH, McIntosh K. Validation of a measure of sustainability of school-wide behavior interventions and supports. Remedial and Special Education. 2019;40(2):67-73.

62. McIntosh K, MacKay LD, Hume AE, Doolittle J, Vincent CG, Horner RH, et al. Development and initial validation of a measure to assess factors related to sustainability of school-wide positive behavior support. Journal of Positive Behavior Interventions. 2011;13(4):208-18.

63. McIntosh K, Mercer SH, Hume AE, Frank JL, Turri MG, Mathews S. Factors related to sustained implementation of schoolwide positive behavior support. Exceptional children. 2013;79(3):293-311.

64. Mercer SH, McIntosh K, Strickland-Cohen MK, Horner RH. Measurement invariance of an instrument assessing sustainability of school-based universal behavior practices. School Psychology Quarterly. 2014;29(2):125.

65. The Board of Regents of the University System of Georgia by and on behalf of Georgia State University and the Georgia Health Policy Center. Positioning for Sustainability: A Formative Assessment Tool – Quick Course. 2011.

66. Markström U, Svensson B, Bergmark M, Hansson L, Bejerholm U. What influences a sustainable implementation of evidence-based interventions in community mental health services? Development and pilot testing of a tool for mapping core components. Journal of Mental Health. 2018;27(5):395-401.

67. Hodge LM, Turner KM, Sanders MR, Filus A. Sustained implementation support scale: validation of a measure of program characteristics and workplace functioning for sustained program implementation. The Journal of Behavioral Health Services & Research. 2017;44(3):442-64.

68. Askell-Williams H, Koh GA. Enhancing the sustainability of school improvement initiatives. School effectiveness and school improvement. 2020;31(4):660-78.

69. Ehrhart MG, Torres EM, Green AE, Trott EM, Willging CE, Moullin JC, et al. Leading for the long haul: a mixed-method evaluation of the Sustainment Leadership Scale (SLS). Implementation Science. 2018;13(1):1-11.

70. Palinkas LA, Chou C-P, Spear SE, Mendon SJ, Villamar J, Brown CH. Measurement of sustainment of prevention programs and initiatives: the sustainment measurement system scale. Implementation Science. 2020;15(1):1-15.
