# Supplementary material for: Evaluation of measures of sustainability and sustainability determinants for use in community, public health, and clinical settings: a systematic review
Source: Implement Sci. 2022 Dec 13;17:81. doi: 10.1186/s13012-022-01252-1 (PMC9746194; doi:10.1186/s13012-022-01252-1)
Supplement: Supplementary file 3 — Additional file 3: Example data extraction fields. [file 13012_2022_1252_MOESM3_ESM.docx]

**Additional file 3: Example of fields included in the data extraction tool**

| Data extraction Item | Response fields |
| --- | --- |
| Publication type | - Published manuscript - Report - Abstract - Technical guide - Thesis - Website - Protocol - Trial Registration - Other |
| Does this measure go by another name? | - Yes - No |
| Please list the other names used for this measure | Open response |
| Was the scale used in this paper an adapted version of the scale? | - Yes - No |
| Please indicate what adaptations were made to the original scale. | - Removed items - Additional items - Changed wording to items - New domains - Deleted domains - Changed response scale - Other |
| Year current version was developed | Open response |
| Study citation | Open response |
| Country study completed | Open response |
| Study design | - RCT - Cross-sectional - Qualitative - Mixed methods - Prospective cohort - Retrospective cohort - Case-control - Other |
| What was the sustainability outcome of interest? | - Determinants of sustainability - Outcome of sustainability |
| Purpose of the article | - Measure development - Psychometric evaluation - Cross-cultural - Empirical study |
| What was the aim of the study? | Open response |
| What was the measure used for? | - Main outcome - Determinants - Confounding - Other |
| Does this measure cover other constructs other than sustainability? | - Yes - No |
| List the other constructs this measure is said to cover in this paper. | Open response |
| Setting that the measure was administered in | - School - Early Childcare - Hospital - General practice - Sporting organisation - Community centre - Other |
| Population who completed the measure. For example, teachers, principals, doctors, allied health | Open response |
| Who administered the measure | - Self-report ‘ - Research staff - Research student - Administration - Clinician - Executive - Other |
| Method of administration | - Pen-and-paper - Online ‘ - Telephone - Face-to-face - Other - NA |
| Number of items | Open response |
| Average time to administer | Open response |
| Number of time-points the measure was administered | Open response |
| Describe the time points (e.g. every 6 months) | Open response |
| Number of domains | Open response |
| How many different response scales are used in the scale? | Open response |
| Study sample size | Open response |
| Response rate (%) | Open response |
| Participant’s characteristics. | Open response |
| Description of the policy, practice or intervention the measure was developed with | Open response |
| Describe how the measure is scored. | Open response |
| Was face validity assessed? | - No - Yes - Unclear |
| Describe how face validity was assessed. | Open response |
| Was target population involved in development of the items | - No - Yes - Unclear |
| Describe how target population were involved in the development of items | Open response |
| Were cognitive interviews used in the development of the measure? | - No - Yes - Unclear |
| Were other qualitative methods used to inform the development of the measure | - No - Yes - Unclear |
| Describe the other qualitative methods that were used. | Open response |
| Was content validity assessed | - No - Yes - Unclear |
| Please provide details of how content validity was assessed and the results of these assessments. | Open response |
| Was measure based on theory or framework | - No - Yes - Unclear |
| What were the theories and/or frameworks used to develop the measure | Open response |
| Was the measure and its items based on other existing validated measures | - No - Yes - Unclear |
| List the names of the other validated measures | Open response |
| % missing values for each item | Open response |
| Were all response options used for each item | - No - Yes - Unclear |
| Was convergent validity measured | - No - Yes - Unclear |
| Were hypotheses for convergent validity stated? | - No - Yes - Unclear |
| How many assessments for convergent validity were assessed | Open response |
| How many assessments of convergent validity were supported? | Open response |
| Detail the results of the convergent validity | Open response |
| Was divergent validity measured | - No - Yes - Unclear |
| Were hypotheses for divergent validity stated? | - No - Yes - Unclear |
| How many assessments for divergent validity were assessed | Open response |
| How many assessments of divergent validity were supported? | Open response |
| Detail the results of the divergent validity | Open response |
| Was known groups validity measures | - No - Yes - Unclear |
| Were hypotheses for known groups validity stated? | - No - Yes - Unclear |
| How many known groups were compared? | Open response |
| How many known groups comparisons were statistically different? | Open response |
| Was structural validity measured | - No - Yes - Unclear |
| What measure of structural validity was used | - Exploratory factor analysis - Confirmatory factor analysis - Item response theory - Principal components analysis |
| Sample size used | Open response |
| Was sample size justified | Open response |
| Results | Open response |
| % variance explained (if relevant) | Open response |
| Was predictive validity assessed | - No - Yes - Unclear |
| Were the other tests being assessed with the measure for predictive validity administered in the future (i.e. after the measure was administered)? | - No - Yes - Unclear |
| Were hypotheses for predictive validity stated | - No - Yes - Unclear |
| How many hypotheses for predictive validity were stated | Open response |
| How many hypotheses for predictive validity were supported | Open response |
| Detail results from predictive validity | Open response |
| Were descriptive statistics for the total measure score and domains provided? | - No - Yes - Unclear |
| Was internal consistency or internal reliability assessed | - No - Yes - Unclear |
| What methods were used to assess internal consistency | - Cronbach’s alpha - Kuder-Richardson 20 - Other |
| Was it calculated | - For total measure score only - For domains only - For total measure sure and domains |
| What was the value for total score (if relevant) | Open response |
| What was the range of values for the domain scores (if relevant) | Open response |
| Was test retest reliability assessed | - No - Yes - Unclear |
| What was the sample size for test-retest reliability | Open response |
| How many days were between the initial test and the retest? | Open response |
| Detail the results of the test-retest | Open response |
| Was responsiveness assessed | - No - Yes’ - Unclear |
| Was the measure administered before and after an implementation intervention? | - No - Yes - Unclear |
| Were floor and ceiling effects assessed | - No - Yes - Unclear |
| What was the % of participants recoding the lowest possible scores | Open response |
| What was the % of participants recoding the highest possible scores | Open response |
| Was concurrent validity assessed | - No - Yes - Unclear |
| Please detail the results of concurrent validity | Open response |
| Was the reading age of the measure reported | - No - Yes - Unclear |
| What grade of reading age was the measure | Open response |
| Was the estimated time to complete the measure reported | - No - Yes - Unclear |
| What was the estimated time for completion in minutes | Open response |
| Is a score calculated for: | - Domains only - Total score only - Both domains and total score |
| Are there any scoring instructions reported | - No - Yes - Unclear |
| Detail the scoring instructions | Open response |
| Are there details relating to how long the measure takes to score | - No - Yes - Unclear |
| How many minutes does the measure take to score | Open response |
| Other psychometric results | Open response |
